# Dual and mutual interaction between microbiota and viral infections: a possible treat for COVID-19

**DOI:** 10.1186/s12934-020-01483-1

**Published:** 2020-11-26

**Authors:** Taha Baghbani, Hossein Nikzad, Javid Azadbakht, Fatemeh Izadpanah, Hamed Haddad Kashani

**Affiliations:** 1 Anatomical Sciences Research Center, Institute for Basic Sciences, Kashan University of Medical Sciences, Kashan, Iran; 2 Department of Radiology, Faculty of Medicin, Kashan University of Medical Sciences, Kashan, Iran; 3 Food and Drug Laboratory Research Center and Food and Drug Reference Control Laboratories Center, Food & Drug Administration of Iran, MOH & ME, Tehran, Iran

## Abstract

All of humans and other mammalian species are colonized by some types of microorganisms such as bacteria, archaea, unicellular eukaryotes like fungi and protozoa, multicellular eukaryotes like helminths, and viruses, which in whole are called microbiota. These microorganisms have multiple different types of interaction with each other. A plethora of evidence suggests that they can regulate immune and digestive systems and also play roles in various diseases, such as mental, cardiovascular, metabolic and some skin diseases. In addition, they take-part in some current health problems like diabetes mellitus, obesity, cancers and infections. Viral infection is one of the most common and problematic health care issues, particularly in recent years that pandemics like SARS and COVID-19 caused a lot of financial and physical damage to the world. There are plenty of articles investigating the interaction between microbiota and infectious diseases. We focused on stimulatory to suppressive effects of microbiota on viral infections, hoping to find a solution to overcome this current pandemic. Then we reviewed mechanistically the effects of both microbiota and probiotics on most of the viruses. But unlike previous studies which concentrated on intestinal microbiota and infection, our focus is on respiratory system’s microbiota and respiratory viral infection, bearing in mind that respiratory system is a proper entry site and residence for viruses, and whereby infection, can lead to asymptomatic, mild, self-limiting, severe or even fatal infection. Finally, we overgeneralize the effects of microbiota on COVID-19 infection. In addition, we reviewed the articles about effects of the microbiota on coronaviruses and suggest some new therapeutic measures.

## Introduction

Mammalian animal species and human are colonized by a group of microorganisms called as microbiota. This group of microorganisms includes bacteria, archaea, fungi, protozoa, helminths, and viruses [1]. These commensal and symbiotic communities of microbes take up residence in almost any part of mucocutaneous areas of the body such as digestive system [2], oronasopharynx and respiratory system [3], urinary system and vagina [4], and all over the skin [5]. There are numerous types of microbiota colonizing in human body. For example, as far as we know there are almost 10–100 trillion microbial cells and more than 1000 different bacterial species that just inhabit in human distal digestive tract [6, 7]. About their origin, plenty of articles suggest that microbial colonization starts immediately after the birth [8]. Formation of the microbiota have several stages. at first in pregnancy and during the physiological changes like weight gain and metabolic or hormonal alterations [9], and then during vaginal delivery, when the neonate is directly exposed to vaginal normal flora. It results in similarities between mother’s microbiota and that of her child. Depending on mode of delivery, there are differences between microbial distribution of the neonates born by cesarean delivery, which is similar to that of skin, and vaginal delivered neonates, microbiotia of whom is similar to that of healthy vagina [8, 10]. The third stage is milking period. Breast milk includes almost 10^9 bacterial cells/L and transfers healthy microbiota from mother to the child, which increase infant’s immunity through competing with pathogens [9–11]. And then, this microbiomes gradually changes to adulthood microbiomes under influence of several factors such as the breed, the family ,geographic and socio-economic factors, diet and nutrition, nutritional supplements, exercises, medications like antibiotics, age, some pathological conditions like inflammatory disorders, diabetes, stress-related factors [12] and finally genetic factors, which play a crucial role in all stages of microbiota formation [13–16]. For example, a study on twins revealed an association between Bifid bacterium and the lactase (*LCT*) gene locus and also a correlation between the host gene *ALDH1L1* and the bacteria SHA-98 [14]. But of note, there are few articles suggesting that the role of genetic factors might be not significant [17]. Many articles demonstrated that every person has special microbial spectrum. Even there are differences between monozygotic twins, so it can be considered as a microbial finger print [9, 14, 18]. The microbiota also have multiple stimulatory or suppressive interactions between themselves, for example, as an suppressive effect, they compete with each other either directly, like nasal *Staphylococcus* species strains which affect other spices via production of some growth inhibitory substances [19], or indirectly, contesting over different sources like nutrient, oxygen and the place of colonization [20]. Additionally, they have stimulatory and synergic effects on each other [1].

## Interactions between microbiota and host

Microbiota have a dual key role in homeostasis, metabolism, immunity, physiologic or pathologic situations like cancers, which can alleviate or aggravate various medical conditions [21]. So here we categorized their effects on various diseases pathophysiology, based on their impacts on different body systems, with a special attention to viral infections.

### Microbiota and the immune system

The microbiota and immune system have communication through several distinct mechanisms and stages. At first the microbiota induces alpha-defensin, secretory IgA and some other AMPs (antimicrobial peptides), thereby they affect innate lymphoid cells, but mainly they affect innate and adaptive immune system via influencing epithelial or macrophage cell receptors such as Toll-like receptors (TLRs) or NOD-like receptors (NLRs). TLRs are involved in normal mucosal immune system development of the intestine, decreasing inflammatory responses and promoting immunological tolerance to the normal microbiota components. NLRs participate in adjustment of IL-18 level, immune response, dysbiosis and intestinal hyperplasia [22–26]. Microbiota antigen binding to these receptors, starts a cascade of signaling pathways leading to producing several antimicrobial substances, like defensine, stimulating several group and subtypes of T cells like T helper 1 and 17 to produce IL-1, IL-2, IL-15, IL-17, IL-22, and IL-23 and also to affect B cells to produce various antibodies [25, 26]. As a result, any alteration in the balance between microbiota and immune system can lead to infections [27], inflammations [28, 29], allergies, several kind of cancers like oral and colorectal cancers [30, 31], autoimmune [32] and endocrine disorders like IBD, diabetes type 1, hepatic diseases which will be explained with more details in ‘’gastrointestinal system disease’’ section. Generally, the microbiome affects dendritic cells, epithelial cells, ILCs, T regulatory cells, effector lymphocytes, NKT cells and B cells [33].

### Microbiota and metabolism

There are many articles on the influence of microorganisms, especially intestinal microorganisms, on body metabolism via comparing to germ-free and normal microbiota colonized animals and humans or through in-vitro studies on gut culture models. Microbiota have enzymes which are not coded in human genome, but they are necessary to fulfil some physiologic tasks or to contribute digestive enzymes to break down substances like polysaccharides, polyphenols and to help in vitamins synthesis. According to this they can regulate body energy balance and cellular metabolism [34–36].

### Microbiota and cancers

The microbiotas can impact upon cancers through different ways. Some of them can induce cancers, some others can prevent malignancies and the rest do not have any effect on caners. Carcinogen microbiotas, contribute in carcinogenesis through three mechanisms. First, they stimulate inflammatory substances release, which can cause inflammation, facilitate cell proliferation and mutagenesis, and activate oncogene and angiogenesis. Second, they inhibit cellular apoptosis via NF-κB activation. Third, they induce carcinogen substances production [37]. On the other hand, microbiota with protective effects against carcinogenesis in the host, prevent malignant transformation by detoxification of nutrient substances, suppressing inflammations, and partake in the regulation of host cells proliferation [38]. For example, low microbial density in upper digestive tract, can lead to cancer-predisposing states in the esophagus and stomach [39]. There are other studies demonstrating association between the microbiota and oral cancer [40], colorectal cancer [41], lung cancer [42] and etc. Also they can be used to diagnose or treat some cancers [38, 43]. Furthermore, probiotics, prescribable foreign bacteria to body, which can settle in mucosal membranes and act against the tumorigenesis along with chemotherapy and radiotherapy [44].

### Gastrointestinal system

Several studies suggest that microbiota play roles in the gut motility and through several mechanisms it can induce or aggravate a wide spectrum of gastrointestinal diseases and conditions, like inflammatory bowel disease (IBD), irritable bowel syndrome (IBS), colon cancer, and antibiotic-associated diarrhea [45, 46]. Also, Christoph A. Thaiss et al. demonstrate that the dysbiosis following the circadian clock disruption can lead to several metabolic and inflammatory diseases like diabetes, inflammatory bowel diseases, and cancer [47–49]. Furthermore, there are articles demonstrating the relation between the gut microbiota and some other diseases unrelated to intestine like cardiovascular disease and blood pressure regulation, hepatic disease (such as chronic liver inflammation, nonalcoholic fatty liver and nonalcoholic steatohepatitis), and pancreatic disease [7, 26, 46, 50]. New studies revealed an association between microbiota, obesity and diabetes [51, 52]. For instance, Martin et al. found that the microbiota and some of their productions can induce signaling messages within the mucosa. Enteroendocrine cells release several hormones like CCK, PYY, GLP-1, GIP, and 5-HT, through which they can adjust metabolic processes like insulin sensitivity, glucose tolerance, fat storage, and appetite. This can explain microbiota’s role in obesity or slimness [53]. They can induce their effects through different mechanisms, like Intestinal permeability, Molecular mimicry or through inducing changes on innate or Adaptive immune system. Moreover, they can be beneficial for diagnosing and treating both type 1 and type 2 diabetes [54–60].

### Nervous system

The microbiota and especially gut microbiota have close mutual relationship with nervous system via plenty of nervous, endocrine, and immune pathways. They can affect CNS and PNS and have a key role in maturation of ENS (enteric nervous system) [61]. For example, they have mutual communication with the brain called as gut–brain axis. Enteric nervous system and the central nervous system both are under influence of them. Thereby, they partake in several CNS diseases like Parkinson’s disease, Alzheimer’s disease [62], schizophrenia [63], multiple sclerosis [64], behavioral disease [65], anxiety and depression [66]. Furthermore, microbiota play roles in ENS formation in newborns [67, 68]. And finally there are studies demonstrating effects of microbiota in sympathetic nervous system, which are involved in the blood pressure regulation and pathogenesis [69]. To conclude the matter, eventually there is a network between central nervous system (CNS), the autonomic nervous system (ANS), the enteric nervous system (ENS) and the hypothalamic pituitary adrenal (HPA) axis. This network links the intestine to nervous system through efferent and afferent autonomic pathways [68].

### Respiratory system

Few years ago, lung was known as sterile parts of the body and even bacterial trace thought to be test mistakes. But new advanced techniques, revealed that lungs have its own special microbiome and is under influence of its microbiome and even microbiomes in other parts of the body such as intestine. Thus, gaining knowledge on the influences of these microbiomes on lungs healthiness and pathology, is in the center of scientist’s attention. A healthy lung microbiota consists of Prevotella, Streptococcus, Veillonella, Fusobacterium and Haemophilus. They come from the upper airways as nose, mouth and pharynx through microaspiration, in which microbiota migrate to lower respiratory tract or lung during sleep. Then they encounter with immune system of the host and thus their balance keeps [70]. Respiratory microbiome formation is under influence of multiple factors, specially in first years of life, consisting of genetic factors, delivery mode, infant feeding, antibiotics administration, medications and vaccination, geographical and seasonal differences [71]. Furthermore, they have roles in some of respiratory diseases. For example, in one hand, in cystic fibrosis (CF), idiopathic pulmonary fibrosis or bronchiectasis bacterial spp diversity in the lower respiratory tracts increases; and in the other hand, some specific bacteria like *Pseudomonas aeruginosa*, *Staphylococcus aureus* or *Burkholderia* spp., have been detected in some specific diseases. In addition, there are connections between gastrointestinal disorders and respiratory system. For example, inflammatory bowel disease (IBD) by the source of microbiota can induce disorders in respiratory system as respiratory diseases which induce disorders in intestine [70, 72–75]. Recent studies suggest that nasopharynx, oropharynx and lung’s microbiome which play key roles in immune system, metabolism and neuroregulation, change in quality and or quantity across several respiratory diseases such as chronic obstructive pulmonary disease (which originates from the gut microbiota) [76], asthma [77], cystic fibrosis [78], pneumonia [79] and also upper respiratory infection [80]. As another example, in one hand, respiratory microbiota tend to be a reason for susceptibility to inhaled toxins or pollutants and in the other hand, some respiratory microbiotas are likely to metabolize inhaled pollutants and even suppress host inflammatory responses to exposure. In addition, their compositions change under each condition and disease [81]. Although there are many studies demonstrating the influence of the gut microbiota on gastrointestinal cancers, few studies have investigated the role of respiratory microbiota on lung cancer; however, new studies has suggested that microbial dysbiosis plays crucial roles in each stage of carcinogenesis via influencing on metabolic, inflammatory or immune pathways [42].

### Microbiota and infectious disease

As time goes on, our understanding increases about the role of microbiota in the host healthy status and their effects on various pathogens. They can inhibit pathogens activities via colonization resistance (competing with pathogens for adhesion to places and nutrients along with secreting microbicidal components like bacteriocin against pathogen), and by empowering the host immune system by mean of contributing to immune cells differentiation, releasing SIGA and inducing proliferation of granulocyte/monocyte progenitors, activation of local innate lymphoid cells, myeloid cells or both pro and pre inflammatory T- and B‐cell responses as noted above [24, 82–86]. Furthermore, they can contribute to extension of secondary lymphoid tissues: such as the gut-associated lymphoid tissue, isolated lymphoid follicles, Peyer’s patches, mesenteric lymph nodes, and splenic white pulps [86]. For instance, inflammation of the gut or antibiotic depletion, alter microbiota’s composition and colonization resistance, leading to enhanced pathogens growth. Recent studies indicate that the commensal microbiota can be used to combat pathogen bacteria specially antibiotic resistant pathogens like *vancomycin-resistant Enterococcus faecium*, *Gram-negative Enterobacteriaceae* and *Clostridium difficile *[24, 82, 83]. Another example is maintaining tight junctions’ integrity by the microbiota to combat Salmonella typhimurium invasion [24]. Also there are studies suggesting probiotics in order to treat pathogen induced diarrheas [87]. In addition, the skin microbiota, includes some bacteriocin-producing strains, that contribute to antimicrobial resistance by producing bacteriocin (an antimicrobial peptide which can also be a signaling molecule to the host immune system). Furthermore, some of them inhibit skin pathogens such as *MRSA* and *C. acnes* and can be used as probiotics for therapeutic purposes via creams or gels [88].

## Microbiota and viruses

Viruses are the most common pathogens in the world. They can infect both eukaryotes and prokaryotes. There is evidence suggesting important mutual interactions between viruses and the microbiotas. They can prevent, suppress or even aggravate viral infections through distinct mechanisms. it’s necessary for a virus to overcome several barriers like tissue specificities, body temperature, mucus barrier and epithelial secretions including IgA and defensins and also microbiotas defending items such as pH, redox potential, lipopolysaccharide (LPS), glycans and finally they are physical barriers for viruses to bind epithelial cells surfaces [89]. Several studies demonstrated that reducing antibiotic administration can improve antiviral responses via balancing microbiota composition. Given that the microbiota exist in the sites which viruses enter to the host, probably they can impact on each other [90]. For instance, the microbiota dictates the development of immune systems via several way such as differentiation and activation of colonic regulatory T cells, which contribute to the maintenance of homeostasis against microbial and dietary antigens [91].

**Fig. 1:**
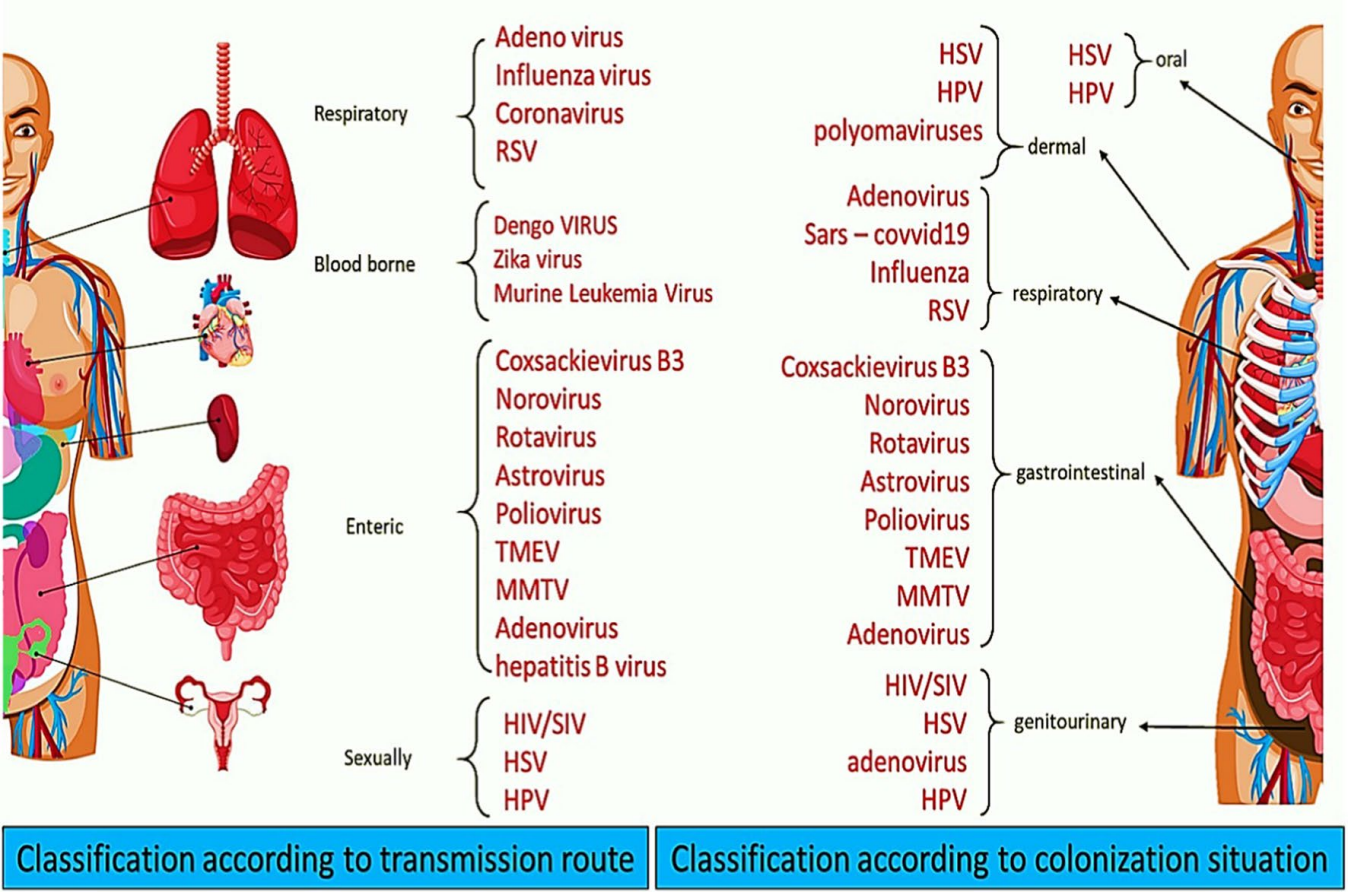
Among the invading pathogens, viruses are one of the most common spp.

The viral infection mechanisms are classified as follow: promotion (which includes direct or indirect mechanisms), suppression (also includes direct and indirect mechanism) and some unknown mechanisms. Generally they have a dual role in viral infections (even in specific virus like HIV): they promote some viral infections like poliovirus and Reoviruses and suppress some other viruses such as MMTV or influenza or even they have dual roles on [90, 91]. There are several specific ways for viruses to enter the host’s body, but in almost all of them, they should cross the mucosal epithelial cells and encounter with microbiota. For example, enteric viruses have fecal-oral transmission so they encounter with the gut microbiota. Sexually transmitted viruses should overcome genital microbiota before entering to the body. Aerosol transmission is a specific way for respiratory viruses which encounters upper respiratory microbiota. And finally blood-borne viruses are directly injected to the body by arthropods and should encounter with both the vector and the disseminated host’s microbiota [86]. To study the effects of microbiota on viral infections we have two choices. First, we can inject antibiotics in mice blood stream as an inexpensive rapid way, but it have some drawbacks, such as antibiotic side effects and antibiotic-resistant species which cannot be eliminated. Second, we can have germ free mice which have been grown in sterile situations since born. This way has also several problems but is a better choice comparing with pervious method [92].

In present study, we will discuss different effects of this microbiota on viral infections via a mechanistic point of view.

**Fig. 2:**
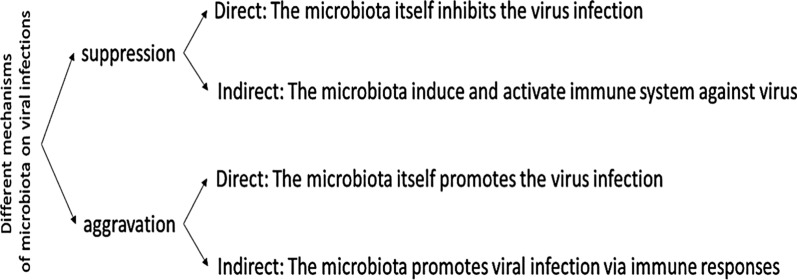
The viral infection mechanisms associated through microbiota

### Different mechanisms against viruses

These mechanisms need more studies but some of them are understood. They divide in three groups including direct binding to the virus, direct antiviral secretion and those resulting from microbiota–host cells interactions. For example, lactic acid bacteria (LAB) are known in industry and their productions (especially exopolysaccharides) have industrial functions. In addition, these secretions have the ability to act against some viral infections like salmonid viruses infections. They are good candidates for therapeutic approaches due to their harmless nature [93]. Lactic acid bacteria (LAB), an antiviral secretion, has been proved by positive signal in immunofluorescence, that can neutralizing vesicular stomatitis virus(VSV) via direct bind to the virus [94, 95].

### Sexually transmitted viruses

#### HIV and HSV

The dominant microbiota in a healthy vagina, mainly consist of Lactobacillus genus, such as *L. acidophilus, L. Fermentum, L. Plantarum, L. Brevis, L. Jenseni, L. Casei, L. Catenaforme, L. Delbrueckii and L. Salivarius*. Their dominance over other pathogenic anaerobes maintain the vaginal health [96]. Also many studies have revealed the impact of altered vaginal microbiota on urogenital infections such as Neisseria gonorrhea, Chlamydia trachomatis, *Trichomonas vaginalis* and finally viral sexually transmitted infections such as human immunodeficiency virus (HIV), human papillomavirus (HPV), herpes simplex virus (HSV) and cytomegalovirus (CMV), which are common current health problems worldwide [97–99]. There is evidence indicating that microbiota and probiotics can protect and even empower the body against them. Probiotics are live microorganisms which have many industrial or medical consumptions. Probiotics have several benefits for the host’s genital system such as maintaining vaginal health, protecting against STIs (Sexually Transmitted Infections) and co-operating with other antimicrobials [100]. Totally the probiotics have similar mechanisms to inhibit pathogens such as HIV. And even they are exactly normal body microbiota which were eliminated in some patients and know we restored them via exogenous sources.

#### Mechanism

There are many studies suggesting that lactobacillus species of microbiota have key roles in HIV prevention. They protect the body against HIV via several mechanisms.

**First**, *lactobacillus* produce H2O2 and acidic metabolites resulted from fermentation and protects the body against HIV and other pathogens like *Neisseria gonorrhoeae*, *C. Trachomatis*, and *T. vaginalis* and even HSV virus. These acidic PH can inactive both virus and immune cells like T cells, monocytes and macrophages which can be a vector or target for HIV [91, 94, 95, 100–106]. Lactic acid and specially l-lactic acid acts against HIV via both direct and indirect mechanisms [86]. Some studies suggest that acidic PH alone cannot inhibit virus and should be provided along with l-lactic acid molecules which has a broad-spectrum HIV virucidal activity. Firstly, it traps HIV-1 in cervicovaginal mucus layer and lowers virus replication nearly 100 times more slowly than that in neutralized mucus, and secondly, it inactivates both HIV-1 and HIV-2 in vitro. Moreover, lactic acid kills BV-associated bacteria which cause indirect inhibition [86].

**Second**, they secret hydrogen peroxide as a virucidal component and also secret anti-viral substances disturbing virus transmission. Furthermore, hydrogen peroxide can combine with several mammalian peroxidases, including myeloperoxidase, eosinophil peroxidase, lacto peroxidase and halide (chloride, iodide, bromide, thiocyanate) in order to make a potent virucidal combination [100].

**Third**, they can directly bind to viruses and disarm them. Lactobacilli and other probiotic bacteria are capable of producing specific HIV inhibitory proteins, whether upon their membrane or in their secretion. For example, two Lactobacillus strains can directly stick to mannose sugar rich “dome” of HIV and lead to neutralizing HIV. Generally, there are carbohydrate-binding proteins on microbiota’s surfaces known as lectins. Mannose *N*-acetyl glucosamine residues are examples of these types of molecules. They have several functions; for example, they competitively stick to carbohydrates on viruses and occupy viral ligands which are necessary for virus to enter immune cells like macrophages or T cells and protect the body from infection. Also, they stick to host infected immune cells like T cells and cut off the infection chain between infected and non-infected t cells. Finally, studies suggest that both glycoproteins and carbohydrates may be involved in vaginal epithelial cell binding and pathogen exclusion [91, 94, 95, 100–106].

**Fourth**, they can improve vaginosis. There are studies which have demonstrated the relation between bacterial vaginosis and HIV infection. BV increases the susceptibly to pathogens and HIV via three manners including causing inflammations, damaging to epithelial cells and putting immune cells and virus in contact and finally reducing hydrogen peroxide and acidic metabolites production [91, 94, 95, 101–106]. There are studies suggesting that Vaginal dysbiosis can be caused by HSV-2 or HIV-HSV-2 coinfection, and conversely BV is associated with increased HSV-2 or HIV infection [107]. First studies about the relationship between BV and STDs was on HIV-1 virus and revealed that woman in different conditions like pregnancy or non-pregnancy, show a positive association between dysbiosis of vaginal microbiota and HIV-1 seropositivity. Also, there is a dose-response relationship between the severity of microbial dysbiosis and HIV-1 serostatus. So that, woman with abnormal microbiota, have more HIV RNA is their vaginal secretion [98]. Vaginal dysbiosis and abnormal vaginal microbiota, can induce the body to produce pro-inflammatory vaginal cytokines such as IL-1β and IL-8, which can lead to a higher risk for HIV infection. Also probiotic lactobacilli can modulate production of these cytokines [98].

**Fifth**, they secret antiviral components. For example, some bacterial species secret bacteriocins which was known as bactericidal components, but recently it has been revealed that they have virucidal effects too. The carbocyclic Iantibiotic labyrinthopeptin A1 and its derivatives have broad anti-HIV and anti-HSV activities. This bacteriocin blocks viral cell–cell transmission between HIV-infected T cells and uninfected CD4 T cells [108, 109].

**Sixth**, they empower he immune system in both vagina and gut; for example, they can release substances like butyrate 38, which supplies energy sources for enterocytes in order to maintain the intestinal mucosal barrier and anti-inflammatory substances, which suppress inflammations and co-infections aggravating HIV infection. Generally, microbial antiviral effects on innate immunity, is based on IL-18, interferon (IFN)-λ, or IL-22 pathways. In one hand, they increase both IL-22 and IL-18 secretion, which lead to more expression in signal transducers and activators of transcription 1 (STAT1) and antiviral genes, and in the other hand they restrain IFN-λ secretions, which contribute to viral pathogeneses [24, 110]. Moreover, some species empower immune system through modulating NF-κB signaling pathways and cytokines, like IFN-ɛ, IL-1α, IL-6, IL-8, and TNFα mRNA, and induce TLR receptors expression, which is important to protect the body against both HSV and HIV[101, 111–113].

It seems that insufficient TLR ligand stimulation after antibiotic exposure was partly responsible for the compromised immune cell function. When TLR agonists were applied during virus challenge in antibiotic-treated mice, both cellular and humoral antiviral responses could be largely restored (Figs. 1 and 2) [65, 67]. Moreover, TLR2 activation by bacterial products produced by the gut microbiota, is necessary for the recruitment of mast cells to sites of viral infection and the further release of cathelicidin, a mast cell-derived antiviral protein (Fig. 2) [68]. However, this situation seems different in young mice, whose gut microbiota has not been completely established. In a hepatitis B virus infection model, TLR4-intact young mice failed to resolve viruses and developed chronic infections, while their TLR4 mutant counterparts exhibited rapid viral clearance, suggesting that an immune-tolerant pathway mediated by TLR4 signaling, was predominant in young mice [72]. Intriguingly, it seems that antibiotic treatment-induced gut microbiota alteration is transient and recoverable, as a more exacerbated disease condition only appears when antibiotics are used during influenza A virus infections; when such treatment ceases before the infection, neither an antiviral immunity defect, nor enhanced viral susceptibility are observed [73].

During the vaccinia virus infection, the commensal microbiota primes virus-specific CD8 + T cells to secrete large amounts of IFN-γ, which critically mediates the corresponding antiviral immunity. In addition, during vaccinia virus infections, the activation of TLR2 by bacterial products is essential for recruiting mast cells to sites of viral infection. These mast cells also contribute to suppressing the viral infection by secreting an antiviral cathelicidin. The gut microbiota is intimately associated with activation of the immune system in HIV-infected individuals [75]. This conclusion is reinforced by another independent study, which showed that the capacity of NKT cells to produce IL-4 and IL-10 in gastrointestinal-associated lymphoid tissues was associated with fewer markers of microbial transmission and less immune activation, a process dependent on the recognition of *Bacteroides* species by these cells [76].

**Seventh**, majority of studies on microbiota and HIV, insist on vaginal microbiota, while there is evidence indicating the role of the male urogenital microbiota on HIV transmission. Semen is an important vector, and seminal microbiota diversity and richness have a key role in HIV transmission. For example, studies indicated that Semen bacterial load affect seven proinflammatory chemokines, like IL-6, TNF-α, and IL-1b, which leads to an alteration in semen viral load [114].

### Herpes simplex virus (HSV) and Cytomegalovirus (CMV)

Because co-infection by HSV can aggravaste HIV infection, anti-HSV properties of these bacteria is important too. In case of HSV, studies demonstrated that Lactobacillus species, set an acidic pH in vagina and produce lactic acid or hydrogen peroxide, which strongly inhibits viral replication and deactivates HSV-2 in the vaginal mucosa. In addition, lactic acid bacterium *Enterococcus faecium* release a small cationic peptide bacteriocin named as Enterocin CRL35 which can block synthesis of herpes simplex virus related protein in Vero cells, thereby inhibiting the late replication stages of both herpes simplex virus types 1 and 2, and can be a good medication because it has no side effect [95, 108, 114–116]. Moreover, there are many studies indicating that maintaining the microbiota compositions, can be a novel therapeutic target to improve health in this patients [117]. Like HIV, vaginal microbiota can directly stick to and trap HSV [98]. Furthermore, *Cytomegalovirus* (CMV) is an important viral pathogen in immunocompromised patients, which can cause many congenital teratogenic infections. Studies suggest that CMV replication and infection with multiple CMV strains can be stopped by normal vagina microbiota [98]. Additionally, Equine herpesvirus 1 (EHV-1) is a virus from herpesviridae family which induces several distinct pathogeneses, like respiratory infections, viral abortion, neurological signs, and neonatal mortality in horses. This study showed that flagellin have reinforcing role in HSV vaccination [118].

#### The gut microbiota role in STD

 The microbiota and probiotics involving in HIV can exist in whether vagina or the gut. As we know, most of immune cells settle in the gut and have several roles in HIV infection. In early stages of infection, the virus depletes CD4+ lymphocytes and dendritic cells of gut associated lymphoid tissue (GALT). It causes several problems including gut barrier dysfunction, microbiota dysbiosis and leaking microbial metabolites to the peripheral bloodstream. The latter is associated with increased levels of proinflammatory cytokines or antimicrobial peptides like (MIP-3, IL-6, IL-8, LL-37, and HNP1-3), and immune cells activation, which in turn can drive HIV progression. Recent studies suggest that probiotic bacteria have a beneficial role in HIV patients during antiretroviral treatment via maintaining microbiota composition, reducing mucosal and systemic inflammation, stimulating natural killer cell, increasing macrophage phagocytic activity, stimulating interferon-γ, interleukin (IL)-12, and IL-18 and improving intestinal barrier [100, 119–121]. In spite of these many studies, which support the idea of beneficial role of microbiota on HIV infection, there are also studies which did not support this [122]. As the last example, vaginal dysbiosis increases in IL-33, which is an alarming compound for epithelial damages, then inhibits T cells migration to vaginal lymphoid tissues, so it can’t secret antiviral interferons and cytokines and thereby promotes HSV infection [121, 123]. Bioengineered probiotics also can have useful effects. For example, commensal bacteria including *Streptococcus gordonii*, *L. lactis*, *L. plantarum*, and *L. jensenii* are engineered to produce a unique 11 kd protein from cyanobacteria called as cyanovirin-N, which can directly stick to mannose sugar rich “dome” of HIV. More over a bioengineered *E. coli* strain in colon can release peptides hybridized with hemolysin A, which can occupy gp41 fusion protein of HIV and causes anal transmission prevention of HIV. And so many other peptides cloned in bioengineered probiotics such as FI-1, FI-2, and FI-3 in *L. plantarum* and *L. gasseri*. And CD4D1D2IgKLC, MIP-1β, and T-1249 in *L. reuteri* RC-14, can occupy gp41 and artificial Antibody to the cellular adhesion in a strain of *L. Casei*, Which blocks transepithelial HIV-1 transmission in vitro [100, 124–126]. Furthermore, In simian immunodeficiency virus (SIV), fecal microbiota transplantation (FMT) treatment improves antiviral peripheral Th17 and Th22 cells activation [91]. By knowing these mechanisms, the author hypothesized that we can focus on any part of them, and by improving or simulating that part we can reach a new treatment.

#### Promotion

 In spite of all the investigations we mentioned above, there are studies suggesting that HIV infected individuals have higher levels of LPS in their plasma, and lipid A, which is a part of LPS, can interact with a peptide derived from the V3 loop of gp120, which is the virus tool to enter the host cells [90]. In addition, SIV which is the HIV ancestor in African primates, can be suppressed by an antimicrobial compound called as glycerol monolaurate. Therefore, it is possible that HIV and SIV utilize the microbiota to develop their pathogenesis as well [90]. Moreover, we should again mention that in spite of these many studies which support the idea of beneficial role of microbiota on HIV infection, there are also studies which have not supported this idea [122]. As the last example, vaginal dysbiosis increases in IL-33 which is an alarming compound for epithelial damages, then inhibits T cells migration to vaginal lymphoid tissues, so it can’t secret antiviral interferons and cytokines and let HSV infection to promote [121, 123]. Furthermore, short-chain fatty acids (SCFA) are microbial components that induce viral infectivity promotion via activation of early lytic cycle of the Epstein Barr virus (EBV) stages, and by inducing expression of viral proteins involved at this stage. What’s more, data suggest that all SCFAs that are histone deacetylase inhibitors, can reactivate herpesvirus, while few of them reactivated the Epstein-Barr virus [127]. As we noted, Flagellin has a beneficial role in herpes vaccinology, but augments HIV-1 entry and promoter activity and increases the production of extracellular virus [128].

#### Human papilloma virus (HPV)

HPV is an oncovirus that causes several cancers, like anal, genital, and oropharyngeal cancers, and elevates the risks of dysplasia and cervical malignancy [129]. HPV is known to induce vagina mucosal infection, mucosal immunity and inflammations via several mechanisms like the induction of interferon, activation of macrophages and NK cells stimulation. Pro-inflammatory cytokines, reactive oxygen species, viral DNA integration, and chronic inflammation during HPV infection changes the vaginal mucosal environment and metabolism, and as a result, alters the vaginal microbiota [130]. For example microbiota studies demonstrated that high proportion of Atopobium, Prevotella, Gardnerella, and in some studies, *Megasphoera *and *L. gasseri* species in women along with CSTs III (microbial dominance of *L. iners*) or with a subtype of CST-IV, probably are HPV-positive and have higher rates in HPV-positive woman, and also have slower enhancement and viral clearance [121, 131]. Also sometimes HPV facilitates other infections, like Chlamydia trachomatis [132]. Moreover there is evidence indicating that microbiota can prevent HPV infection by producing d-lactic acid, producing hydrogen peroxide, and blocking HPV adherence to vaginal epithelial cells by forming a microbial physical barrier [96, 114]. Several studies demonstrated the association between vaginal microbiota, HPV infection, and clinical cervical neoplasia. In such a way that dysbiosis, by higher abnormal rates of *Gardnerella vaginalis *and *Lactobacillus gasseri* and a lower abnormal rates of *Lactobacillus crispatus*, *Lactobacillus iners* and *Lactobacillus taiwanensis*, induces HPV infection and HPV-dependent neoplasia of cervix via producing carcinogens like nitrosamines; while opposite situations can accelerate HPV clearance and improvement. There are many other studies indicating the role of microbiota on HPV-associated cancers with just few differences in bacteria they found [96, 130, 133, 134]. However, generally the mechanism of HPV immunological pathways on vaginal microbiota are not completely understood and need more investigations [121].

### Blood born viruses

#### Dengue virus

Dengue virus (DENV) is an insect transmitting virus, which infects 390 million people per year. It transmits via an insect vector called *Aedes aegypti *[135]. These insects, in some cases, have a commensal bacteria called as Wolbachia, which cooperate with other microbiota and reduce insect susceptibility to dengue virus indirectly via inhibiting virus replication by inducing more expression in antimicrobial peptide producing genes, controlled by Toll-like receptor (TLR) immune pathways [135, 136]. Toll pathway repression via silencing of MyD88 gene, resulted in increased dengue titers. Totally, Wolbachia induces its effects through basal level stimulation of the Toll immune pathway [86]. Moreover, the insects administrated antibiotics, showed higher levels of virus titers [90]. Therefore, it came to our mind that maybe it would be possible to use the microbiota of other species such as Wolbachia, for therapeutic purposes in humans as probiotics. Also at least we can produce their metabolites via bioengineering, but it take more exploration to understand their actual mechanisms.

#### Murine leukemia virus

Murine leukemia virus (MuLV) is a enveloped gamma retrovirus virus of the Retroviridae family, and are responsible for leukemia/lymphomas in mice. It is a blood born virus that transmits vertically through milking or parturition and other routes like birth bites and scratches [86]. The microbiota increases GF (Germ-fee) MLV infection in mice via inducing lymphoid cells division and proliferation and also facilitating viral replication. In a study, it was revealed than GF mice are more resistant to MLV-induced leukemia than SPF mice, due to lymphoid cells stimulation of the microbiota [137]. But there are conflicting studies showing positive, negative, and neutral effects of microbiota on this virus and MLV infection in GF mice, probably due to its strains or experimental mistakes. For example, some MuLV viruses contain a contaminating lactate dehydrogenase levating virus, which exerts systemic lymphocyte activation [91, 138].

#### Lymphocytic choriomeningitis virus

Lymphocytic choriomeningitis virus (LCMV) is an enveloped, RNA virus belonging to the Arenaviridae family, which can produce both acute and chronic infection in mice [139]. The microbiota reduces LCMV-specific CD8+ T cell responses and IgG antibody titers which lead to antiviral responses and lower infection duration. Mechanistically, antibiotic-treated mice CD8+ T cells, express more inhibitory receptors and less effector molecules, which means that lack of microbiota, can lead to T cell exhaustion. Unlike antibiotic-treated mice, macrophages in SPF mice demonstrate higher antiviral responses. The dysbiosis in antibiotic-treated mice reduces innate and adaptive immune responses against LCMV infection [137].

### Enteric viruses

As noted above gastrointestinal or enteric viruses have fecal-oral transmission. Viral infections and intestinal microbiota have distinct effects on each other. The microbiota can suppress or promote viral infections and conversely viral infections can induce eubiosis or dysbiosis and even some of them, like Murine norovirus (MNV), can replace beneficial roles of the microbiota in germ-free mice [140]. The most common enteric viruses like rotaviruses, noroviruses, and astroviruses are non-enveloped RNA viruses that infect the gastrointestinal tract and lead to severe childhood diarrhea and gastroenteritis. More over reoviruse, mouse mammary tumor virus (MMTV) and poliovirus are also enteric viruses in nature. Even some systemic viruses like influenza and Coxsackievirus B3 viruses have an intestinal colonization [7].

#### Rotavirus

Rotavirus (RV) is a nonenveloped, double-stranded RNA virus from the Reoviridae family and is one of the most common leading causes of death in 1–5 years old children due to pediatric diarrhea, with an estimated 200,000 deaths per year in whole of the world [137]. Studies suggest that there is a dual connection between the microbiota and rotavirus: rotavirus infections can induce changes in Bacteroides genus composition, and microbiota and probiotics have been demonstrated to be effective therapeutic agents in Rotavirus infection [141]. Probiotics, like *Lactobacillus rhamnosus*, can reduce both viral diarrhea duration and diffusion. For example, a microbial soluble secretion, blocks rotavirus infection in the gut via preventing rotavirus attachment by altering intestinal epithelial cell surface glycans [137]. In addition, Rota virus flagellin activated TLR5 pathway have influence on dendritic cells and leads to (IL)-22 release. Also, it increases NLR Family CARD Domain Containing 4 (NLRC4) dependent IL-18 discharge. IL-22 maintains the epithelial cell and its proliferation in normal status, while IL-18 eliminates infected epithelial cell via apoptosis. Hence, this pathway immediately removes rotavirus (RV) infection and accelerates RV clearance [142]. Moreover, a synbiotic combination of galacto-and fruct-ooligosaccharides mixed with Bifidobacterium breve, have a negative effect on RV virus via increasing the production of IFNγ, IL-4, TNFα, and TLR2 expression. In conclusion, we can assume that, it indirectly affect RV through inducing effects on immune responses, such as lowering immune tolerance and enhancing the mucosal defense [143]. However there are studies demonstrating positive effects of microbiota on rotaviruses; for example, human milk oligosaccharides contains probiotics like *Bifidobacterium*, which lead to RV infection promotion via immune response. So that antibiotic treatment decreases infection by suppressing RV entry via improving IgA-producing cells. Also lack of microbiota in antibiotic treated or GF mice, reduced the level of rotavirus antigen, delayed infection and decreased infectivity [144]. Furthermore, increasing Proteobacteria and decreasing Bacteroidetes amounts in vancomycin treated human, promotes RV vaccine immunogenicity and RV shedding [145, 146]. In addition, commensal microbiota also inhibits the activation of antiviral humoral responses via decreasing virus-specific antibodies amounts including IgA and IgG in serumic and IgA in fecal samples. Antibiotic treatment maintains virus-specific antibody-secreting cells in the intestine and restores microbiota, and administration of low doses of dextran sodium sulfate leads to enhancement in production of rotavirus-specific antibodies and this is why microbiota have positive effects on rotavirus [91, 147].

#### Norovirus

Norovirus is a non-enveloped, single-stranded RNA, enteric virus from the Caliciviridae family and transmits through fecal-oral route and also they are foodborne viruses. Norovirus is a common cause for viral gastroenteritis and the most common cause of severe childhood diarrhea in developed countries. There is no treat or vaccine for norovirus yet and diarrhea, vomiting, nausea, stomachache, and short term fever are its symptoms [137, 148, 149]. The Norovirus genus classifies in seven Geno groups (GI–GVII). For example, GI and GII viruses are responsible for human infections, while there are strains of murine noroviruses (MNV), which are not present in GV classification. Murine noroviruses have no effect on humans, while are good candidates for experimental investigations. Also in both human and murine strains we can see similar positive or negative effects caused by microbiota [150].

#### Suppression studies

Demonstrated a positive relation between the density of *Ruminococcaceae* and *Faecalibacterium *spp. and anti-NoV antibody titers, so that, it implies the possibility that these bacteria can have protective roles against NoV infection. Previously, *Faecalibacterium *spp. were known as an anti-inflammatory bacteria, but the mechanism is unclear. Moreover, in case of MNV, poly-g-glutamic acid (g-PGA), a Bacillus spp. secreted component, protects mice from MNV-1 infection via regulating MNoV infection by increasing interferon-b (IFN-b) signaling pathways [151]. Although most of retinoic acid dependent studies investigated MNV, there are some studies revealing that retinoic acid treatment reduces the risk of HNoV infection [152]. The Lactobacillus bacteria suppress murine norovirus (MNV) replication via expression of interferons, like IFNβ and IFNγ and lower infection duration. Although retinoic acid has antiviral effects alone, but it will be stronger along with the microbiota. MNV-1 reduces *Lactobacillus *spp. and retinoic acid or vitamin A restore them. Therefore, we can draw the conclusion that the antiviral effects of vitamin A and retinoic acid, are due to Lactobacillus genus interferon production [153]. Moreover, a study revealed that vitamin A induces antiviral effects via modulation of gut microbiota, such as Lactobacillus spp., which upregulates IFN-β and IFN-γ expression in MNV-infected RAW 264.7 cells. Then IFN-γ promotes adaptive immunity responses to MNV infection. Other studies provided more confidence for this idea; for example gram negative microbiota can produce LPS, which induce IFNs secretion and lead to inhibit the replication of MNV [154].

#### Promotion

According to many studies, antibiotic treatment inhibits viral norovirus infection which shows the role of microbiota. In case of human norovirus, most of the studies suggest that human norovirus directly sticks to both microbiota and the host surface components like histo-blood group antigens (HBGA) via VP1 capsid protein. Nevertheless, there are studies indicating that human norovirus can bind to the microbiota which have no HBGA. So we can conclude that it may be due to different strains or some other unknown attachment components [149, 155]. In conclusion, HBGA attachment to the virus, leads to stabilizing (especially thermostabilization) viral particles by bacterial ligands, then glycan-bound viral particles facilitate viral attachment to target cells receptors and host-to-host transmission as direct mechanisms. Additionally, they can induce indirect effects via affecting the host immunity and preparing an immune tolerance, which leads to more viral replication and also leads to promoting viral recombination (149, 155]. Another study revealed that in an in vitro B cell culture system, the microbiota producing HBGAs have an important role as a cofactor for HNoV replication and even administration of bacterial HBGAs alone was sufficient [155, 156]. In addition, antibiotic treatment indirectly promotes expression of antiviral receptors such as Stat1 and Irf3 for cytokines like IFN-λ [91]. The microbiota can induce epithelia lesions and damage epithelia defense in SPF mice, while have no effects on GF mice. So it causes intestinal inflammation via IL-10 induction and leads to more viral transmission and replication [157]. In addition, vesicle-cloaked virus clusters (VCVCs) are a group of non-negligible viral populations in stool and have a more infectivity than free viruses [158]. For instance, they contain both norovirus and poliovirus and also other viruses, like poliovirus which increase co-infection and recombination between different viral strains. Microbiota can mediate viral clustering directly or indirectly which enhances viral infectivity [151]. Also, the microbiota modulates HNoV infection via Bile acid regulation. HNoV capsids directly stick to bile acids and increases HNoV replication in a dose-dependent manner via facilitating their binding to HBGAs. This shows that the bile constituents, which act as HNoV cofactor, require bacterial metabolism for their action [151].

#### Astroviruses

They have been reported to account for approximately 2–9% of pediatric cases of gastroenteritis worldwide. Colonization of immunodeficient mice with a murine astrovirus (STL5), induces intestinal lambda interferon (IFN-l) [7].

#### Reovirus

Reovirus is a non-enveloped, double-stranded RNA virus from the Reoviridae family, which is an enteric virus colonizing the gut but it is usually asymptomatic [7]. Reovirus replication and pathogenesis reduces in antibiotic-treated or GF mice. So it shows that microbiota promote reovirus replication and pathogenesis although the mechanisms are not very clear [137]. In both poliovirus infection and reovirus infection, the microbiota elevate the virus stability and specially thermostability via direct interaction between Gram-negative and Gram-positive bacteria. For example, there are several studies indicating that antibiotic treatment and microbial depletion before these viral infections reduces virus’s virulence [91]. In conclusion, reovirus can cooperate with both Gram positive and Gram negative bacteria via influencing directly on bacterial outer envelope components and also can loosely attach to Gram-positive or Gram-negative bacteria using LPS or GPs to enter their target cells [159]. But LPS alone can’t cause these effects and needs other components existing on Gram negative bacterial cell surfaces to complete its work. To confirm this fact, in a study reovirus incubated with E. Coli showed more stability in comparison to LPS alone [159].

#### Poliovirus

Poliovirus is an enteric non-enveloped single-stranded RNA virus from the Picornaviridae family, which can be transmitted through fecal-oral route. It spends some days replicating in the gut and then migrates to central nervous system, where its pathogenesis occurs and leads to paralysis. In addition, Poliovirus is a human pathogen which needs a human poliovirus receptor (PVR) and in order to experiment, we have to use PVR-transgenic mice [7, 160]. The microbiota aggravate poliovirus replication and pathogenesis via both direct and indirect mechanisms. In one hand both gram-positive and Gram negative microbiota bind their polysaccharides to poliovirus to stabilize the virion by preventing premature RNA release. For example, peptidoglycan and LPS released by microbiota which have amounts of N-Acetyl glucosamine, bind poliovirus and induce viral thermostabilization even at temperatures above 40 °C. In the other hand, LPS facilitates poliovirus binding to its cellular receptor and target cells [137]. Also, poliovirus incubation with the microbiota before the infection initiation, increases the genetic recombination and co infection possibility between different phenotypes like drug guanidine hydrochloride, while resistant to high temperature (DrugSTempR) or resistant to guanidine hydrochloride, while sensitive to high temperature (DrugRTempS) [91]. Furthermore, it is interesting that even UV-inactivated microbiota incubated with poliovirus can induce viral stabilization because of their bacterial surface polysaccharides (LPS and peptidoglycan), which can do their job independently [91]. More confidence for this fact prepared by using a mutant poliovirus, which have lower binding tropism to LPS via putting a single amino acid in the viral capsid protein VP1-T99K. The mutant viruses had equal replication, attachment, and pathogenesis comparing with the wild-type in vivo, but after passing through the gut had lower stability in mouse feces compared to their wild-type [161].

#### Mouse mammary tumor virus (MMTV)

Mouse mammary tumor virus (MMTV) is an enveloped enteric virus from the Retroviridae family, which can be transmitted vertically through infected mother’s milk to the children and causes gastrointestinal infections or transmits. Although TMEV is an enteric virus in nature, its pathogenesis and replication is in neurons and leads to multiple sclerosis in mice [7, 162]. Studies revealed that microbiota components like lipopolysaccharide (LPS) aggravate the TMEV replication and transmission and disease by increasing central nervous system inflammations via TLR4 activation, which is a pattern-recognizing receptors (PRR) for LPS [137]. In conclusion, MMTV utilizes LPS to stimulate TLR4 expression in order to induce IL-6, which also leads to increase immunosuppressive cytokine IL-10 amounts as a key cytokine to modulate the immunoregulatory roles of T-reg cells and prepare an immune tolerance. This cascade finally helps the virus to escape from immune system through TLR4/MyD88 pathway. As a result, even the MMTV isolated from mice without LBPs cannot capture LPS and stimulate TLR4 [138]. In addition, In a follow-up study it was revealed that LPS receptor contributes to viral envelope formation and promotes viral diffusion [162]. It means that MMTV utilizes LPS binding proteins such as CD14, TLR4, and MD-2 into its viral envelope for next generations [149].

#### Adenovirus

 Adenoviruses infect humans by respiratory and enteric routes. Human adenovirus is a non-enveloped, double-stranded DNA virus of the Adenoviridae family. The microbiota can produce defensins which is an antiviral component against herpesviruses, human *papillomaviruses, polyomaviruses, orthomyxoviruses*, and retroviruses. Defensins like alpha defensin 5 directly bind to human adenovirus and limit viral replication in cultured cells by preventing uncoating in endosomes, but the exact mechanism remains unclear [6, 137].

#### Hepatitis B/C virus

Hepatitis B viral infection is a chronic persistent infection, which can be suppressed by the gut microbiota via interaction with Ab secreting B cells. The microbiota decrease hepatitis B virus (HBV) e-antigen (HBeAg) amounts after each fecal microbiota transplantation (FMT) therapy [91].

#### Coxsackievirus B3

Coxsackievirus B3 (CVB3) is a non-enveloped single-stranded RNA virus belonging to the to the Enterovirus genus of the Picornaviridae family [163]. At first, antibiotic treatment reduces systemic poliovirus and CVB3 titers, which means that the microbiota promotes CVB3 infection, then it reduces both CVB3 infection and dissemination kinetics. It has been shown that antibiotic treatment decreases CVB3 titers in both human cecum and GF mice, and the reason for that is antibiotic treatment increases mural innate immune responses and antiviral activity against both DNA and RNA viruses [137, 164].

#### Respiratory viruses

The respiratory tract is one of the most proper sites for microbial incoming and colonization, which may cause asymptomatic, mild, severe and even fatal infections. There are many studies on enteric microbiota, but fewer studies about respiratory microbiota have been conducted. Microbiota have higher density in upper respiratory tract and gradually, it decreases in lower respiratory tracts. Distal tracts and lung seem to be isolated from microbes. But even some studies revealed that the lungs have also certain microbiota, and this fact that they are free of microbiota comes from limitation in culturing, sampling and lack of advanced techniques to measure them. These microbiota in both lungs and respiratory tracts, have also key roles in some respiratory diseases such as asthma, cystic fibrosis, or chronic obstructive pulmonary disease [165–169]. A study by Leung et al. demonstrated that the upper respiratory tract microbiome consists of Firmicutes, Proteobacteria and Actinobacteria [170]. Furthermore, a study concluded that the healthy human lung does not include a consistent distinct microbiota, but interestingly it includes a special spectrum of microbes similar to the upper respiratory tract’s microbiota. This insists on significance connection between the upper and lower respiratory tract microbiota [171]. Moreover, viral infections, the most common pathogens responsible for the respiratory system infection, along with their pathogenicity, simultaneously alters respiratory microbiota to the extent of disease severity, which implicates these communities in the immune activation status of these patients [172], and here we provided a review about several respiratory viruses and their interactions with both intestinal and pulmonary microbiota mechanistically, which may leads to prepare new probiotic treatments or medications.

#### Human rhinovirus

Human rhinovirus (RV) is the major cause of the common cold and a frequent cause of respiratory tract infections with considerable morbidity and mortality in patients [173, 174]. Toivonen et al. found a strong association between HRV colonization and changes in the microbiota [175]. Korten et al. demonstrated that there is an active interaction between HRV infections and the respiratory microbiota in early life. To be more precise, HRV symptomatic infections have interactions with a short-term alteration in microbial density and diversity. Also, more common symptomatic HRV infections have a long-term influence on microbial diversity at the end of the first year of life. In addition, a study on adults revealed a lower diversity of microbiota during symptomatic HRV colonization [176].

#### Influenza A virus

Influenza is an acute respiratory disease that is caused by influenza virus. Influenza is classified as contagious diseases that infects the host epithelial cells [177]. Influenza virus has two major surface Glycoproteins called hemagglutinin (HA) and neuraminidase (NA). The HA makes a fusion between virus and target cell receptor (sialic acid), and the NA will destroy and cleave the a-ketosidic linkage between terminal sialic acid and an adjacent sugar residue [178]. Influenza virus has 3 types; A,B and C. Naming is based on antigenic diversity of proteins in nucleus and matrix of the virus. Influenza A virus (IVA) is the most pandemic type [178]. IVA causes an acute infection in respiratory tract. Researches have shown a low level qualitative changing in lower respiratory tract microbiota composition during IVA infection [179]. Alterations such as increase in a bacterial load, can raise the risk of secondary bacterial infection, not only in respiratory tract, but also in other sites, like intestine [180]. Thus, IAV infection can affect intestinal microbiota community too. IAV destroys mucus layer integrity, therefore can impress the immune responses [179]. Also IAV may cause gastroenteritis symptoms, like diarrhea [181]. People with influenza infection may experience some gastrointestinal inconvenience and disorders. It can be secondary to previous active infection in that site, or can be result of swallowing infectious particles from respiratory tract, which will be transferred to gastrointestinal tract and make the symptoms [182]. The microbiota can affect influenza virus by a way which is associated with a significantly decreased antiviral immune response in the intestine. So antibiotic treatment can have negative effects in H7N9 influenza virus infected propels [183–185]. Influenza infected mice which take antibiotic treatments, have lower level of T cells, and the T cells also have lower capacity to produce TNF-ɑ, MIP-1ɑ, IL-2, and IFN-γ; besides, antibiotic treatment have some effects on pulmonary macrophages. The gut microbiota dysbiosis which is caused by antibiotic treatment, will decrease the level of Mx1, Ifnb, Il1b, Tnfa, expressions as antiviral genes in macrophages of pulmonary system during influenza infection [111]. Antibiotics can reduce the variety of intestinal microbiota [186] and there is a relationship between influenza virulence and gut microbiota variety [113]. This gut microbiota variety reduction is what that exactly happens in H7N9 infected persons, and also an increase in Escherichia coli and *Enterococcus faecium* colonization have been revealed in these persons [183].

A study revealed the role of nasal and pharyngeal microbiota before influenza infection, on duration of symptoms and also the viral shedding via impacting on viral transmission. For example, a variety of bacterial communities, prolong the duration of viral shedding and cause earlier occurrence of the associated symptoms like Neisseria logotype which cause earlier signs of infection but longer durations of symptoms [187] Microbiota restoration treatment reduced the incidence of Enterogenous secondary infection, but not exogenous respiratory infection [188]. The Microbiota can produce anti-viral components, which have direct or indirect effects on the virus. For example, the microbial derived LPS, can bind directly to influenza to reduce its stabilization [189]. As another example from a different study, it has been revealed that *S. Epidermidis*, as a nasal microbiota, presents both in vitro and in vivo anti-influenza virus activity via secreting a giant extracellular matrix-binding protein (Embp) [190]. Furthermore, the microbiota can induce immune responses. For example, desaminotyrosine (DAT), which is produces by *Clostridium orbiscindens* in mice, prevents influenza infection and its lung immunopathology via inducing type I interferon and activating this signaling pathway [191]. It should be noticed that, in non-influenza virus infections like HIV infection, there are several mechanisms for lactic acid bacterium *Enterococcus faecium*, by which it directly traps viruses [91]. Another study concluded that the microbial antiviral effects are due to microbial signals, which establish the innate immune system activation threshold. They affect the infection via reducing IFN activation genes expression in macrophages. So that ABX mice(mice underwent antibiotic treatment) macrophages have lower ability to control type I and type II IFNs [111]. The type I of IFN system (IFN-I) is a first line of the anti-viral immune responses [192]. Hence, basically the immune system produces this component in influenza infection too. One research on mice has shown that during influenza infection, IFN-I can make some changes in gut microbiota as well. Evidence shows that lung induced IFN-Is due to changes in gut microbiota, can cause an increase in Salmonella growth in the inflamed gut and can induce its systemic propagation to some other sites. This gut dysbiosis has been shown with the decrease in commensal obligate anaerobic bacteria community and the increase in gut proteobacteria community [182]. Generally, intestinal microbiota and probiotics such as *Lactobacillus paracasei* and *Lactobacillus plantarum* elevate both pro-inflammatory cytokines like IL-33, IL-1α, IL-β, IL-12, and IFNγ, and also they increases the presence of innate immune cells in the lungs such as NKs, macrophages, and dendritic cells during influenza infection. They also increase IL-10 and lead to both reduction in the inflammatory response in the lungs and adjustment of the antiviral response [138, 193]. A study on mice demonstrated that probiotics can be of benefit in influenza infection. It was shown that mice which had taken Bifidobacterium breve YIT4064 had higher level of anti-influenza virus IgG in their serum in comparison to those which had not taken probiotics [194]. Another study demonstrated the higher level of IL-2, IL-12, IL-15, IL-21, IL-18, and IL-1Β expressions in lungs and peyer’s patches of mice which were treated by some probiotics like *Bifidobacterium longum *MM-2, *Lactobacillus casei* Shirota, *Lactobacillus pentosus* S-PT84, *Lactobacillus plantarum* 06CC2, *Lactobacillus rhamnosus*, *Lactobacillus paracasei*, *Lactobacillus gasseri* TMC0356, *Lactobacillus brevis* KB290, *Lactobacillus acidophilus* L-92, L. Plantarum DK119 or *Lactobacillus fermentum* CJL-112 and there was a depletion in IL-6, IL-4, IL-5, and IL-10 levels as immune responses which was mediated by Th1 in probiotic treated group of mice [195]. Influenza A virus-infected macrophages, secrete some cytokines which are able to induce IFN-γ synthesis in human T cells [196]. Moreover, an study showed that when an influenza infection causes lung injury, some of lung-derived CCR9+ CD4+ T cells migrate to small intestine, and start to produce IFN-γ. IFN-γ in turn can mediate the intestinal microbiota and cause a microbiota alteration during the viral infection. Microbiota alteration affects epithelial cells in the intestine and thereby stimulates IL-15 production which promotes Th17 cell polarization through the PR8 infections (respiratory infections). Then the number of Th17 cells significantly increases in the small intestine and it neutralizes IL-17A, by which decreases the intestinal damages [181]. The gut microbiota reduction in influenza infection, can impede dendritic cells (DCs) migrations from lungs to lymphoid tissues. So it diminishes CD4+ and CD8+ T cell expansion and B cell differentiation [113]. Other study showed the microbiota’s influence on the T cells differentiation and expansion as well [29]. Furthermore, another study by Ichinohe et al. , revealed the ability of intestinal microbiota to regulate both innate and adaptive immune responses against influenza virus infection, as a result of low proliferations of lymphocyte such as CD4+ and CD8+ T cells in the lungs. This problems get worse with local or distal injection of Toll-like receptor ligands [195]. But previously it had been found that Staphylococcus aureus provokes the recruitment of peripheral CCR2 + CD11b + monocytes and their subsequent maturation into M2 macrophages, via stimulating TLR2 signaling pathways in influenza infection which leads to enhancing acute lung injury [197]. It should be noticed that unlike most of the studies supporting inhibitory roles of microbiota on influenza infection, there are studies suggesting that the microbiota may have no regulatory effects on antiviral responses. For instance, previous studies revealed that although neomycin has inhibitory effects on several viruses like HSVs and influenza A virus, its antiviral activity was due to neomycin itself. It activates TLR3 in some dendritic cells and leads to increasement in the IFN-stimulated genes expression, which neomycin itself is responsible for, and it isn’t related to the microbiota [138, 170]. Chaban et al. and Leung et al. in two distinct studies revealed that *Proteobacteria*, in particular the *Enterobactericeae* and *Moraxellaceae* families, are more frequent in influenza infected patients rather than normal non infected peoples. In conclusion, these evidence suggest that the Proteobacteria species may have been correlated to secondary infection in influenza A infection [170]. Evidence revealed the effects of microbiota restoration to GF mice on lethal influenza virus infections in mice. They decrease inflammation via IL-10 and IL-13 production which leads to decrease mortality [198]. As noted in "HIV virus’’ section, LPS activation of TLR4 leads to acute systemic sepsis, chronic inflammatory diseases particularly in viral infections such as influenza and HIV infection. Influenza-induced lethality is due to the TLR4-stimulating effects of TLR4 agonists damage-associated molecular patterns (DAMPs) which can stimulate monocyte-derived dendritic cells, inducing proinflammatory cytokine secretion and FP7 prevented DC activation by HMGB1 [199]. The beneficial therapeutic role of flagellin in viral infections with species like rotaviruses and influenza viruses and even herpes viruses have been proven by several studies. For example, a study revealed therapeutic role of flagellin administration, which can decrease viral load in the lungs of an influenza A virus infected mice [200]. Accompaniment of flagellin with influenza vaccine or inactivated influenza viruses leads to higher IgA and IgG titers against influenza virus via TLR5. These evidence, indicate the reinforcing role of flagellin as a therapeutic component and a potent mucosal adjuvant [201]. Moreover, flagellin have reinforcing role in HSV vaccination [118].

#### Respiratory syncytial virus

Respiratory syncytial virus (RSV) is the most common ethiology behind lower respiratory tract hospitalization and mortality in younger age people worldwide. Distinct microbiota have different effects on respiratory syncytial virus bronchiolitis through regulating the systemic immune pathways. Streptococcus and Haemophilus influenzae suppress and Staphylococcus aureus promotes RSV viral infection severity. They regulate proinflammatory genes expression to activate immune cells such as macrophages and neutrophils via an unknown mechanism [202]. Another study by Persia et al. in 2019, reveled an important cross-correlation between nasal microbiota (assessed as β diversity) and white blood cells level, Lambda3 and Beta IFN genes expression and Th1/Th2 response [203]. Moreover, *Corynebacterium pseudodiphtheriticum* a respiratory microbiota, controls antiviral TLR3 response against Respiratory Syncytial Virus (RSV) via increasing T-cell subpopulations, which induces TNFα, IL-6, IFNγ, and IFNβ secretion [204, 205]. Munoz and colleagues revealed that although RSV immunoprophylaxis is clinically beneficial for RSV infection specially during the highest respiratory vulnerability period, but it might cause long-term alteration in the respiratory microbiota [206]. The last interaction between RSV and the microbiota hyaluronic acid (HA) is a bacterial component, which have roles in inflammation, tumors, and viral infections caused by viruses like RSV. HA can be produced by several bacteria including *Streptococcus* spp., *E. fecalis*, *E. coli*, *L. lactis*, *B. subtilis*, *Agrobacterium *spp., while several other bacteria including Streptococcus A, B, C, and G, S. Pneumoniae, *C.* *Perfrigens *break down HA via producing hyaluronidase [207].

#### Ebola virus

Microbial colonization on the skin, also known as the skin microbiome, strengthens the skin’s defense against potentially pathogenic organisms. keratinocytes obtained from normal skin, attach and spread through binding to the thrombospondin (TSP, also referred to as THSD) family of proteins; This interaction may play a role in initiating cell-mediated immune responses in the skin by releasing cytokines and stimulating the expression of TSP proteins (Varani et al., 1988) to facilitate the movement of immune competent cells (Baker et al., 2003). TSP promotes keratinocyte attachment and spread, and may play an important role in maintaining the normal growth of the basal cell layer.

The discovery of microRNAs (miRNAs) in Ebola virus implies that immune escape, endothelial cell rupture, and tissue dissolution during Ebola virus infection, are a result of the effects of Ebola virus miRNAs. keratinocytes obtained from normal skin, can attach and spread through expression of the thrombospondin family of proteins, playing a role in the initiation of cell-mediated immune responses in the skin. Several miRNAs have been shown to bind the 3′ untranslated region of thrombospondin mRNA, thereby controlling its stability and translational activity. In this study, we found that short RNA sequences may act as miRNAs from Propionibacterium acnes using a practical workflow of bioinformatics methods. Subsequently, we deciphered the common target genes. These RNA sequences tended to bind to the same thrombospondin protein, THSD4, emphasizing the potential importance of the synergistic binding of miRNAs from Ebola virus, Propionibacterium acnes, and humans to the target. These results provide important insights into the molecular mechanisms of thrombospondin proteins and miRNAs in Ebola virus infection.

Currently, no specific therapies have been shown to be effective for the treatment of EHF. Thus, only supportive therapies are typically given in an attempt to overcome the infection; however, the fatality rate remains high. EBOV can spread through direct skin contact. Recent studies have proposed that EBOV infection is a result of the action of EBOV miRNAs (Liang et al., 2014). As previously mentioned, microbes in the skin microbiome, such as P. acnes, potentially play a vital role in protecting the host.

Recent studies have shown that EBOV infection could occur through the activities of EBOV miRNAs (Liang et al., 2014). As mentioned above, *P. acnes* may play an important role in host protection. Thus, in this study, we established a bioinformatics approach to detect short RNA sequences in *P. acnes* based on sequence alignment; accordingly, our findings demonstrated that these *P. acnes* sequences may act as miRNAs and protect humans from EBOV infection by regulation of the expression of their common target gene THSD4 [208, 209].

### The impacts of viral infection on the microbiota (eubiosis or dysbiosis)

#### SIV/HIV

 HIV infection damages gut-associated lymphoid tissues (GALTs) and reduces Th17 cells number, that modulate the gut bacteria. By several way HIV infection can cause alternation in microbiota composition, like inability of the both innate and adaptive immune system to qualify the commensal bacteria, like inability in CD4 T cells defense. Another reason can be existence of inflammation tolerant microorganisms that are the result of chronic inflammatory situation. The intestinal microbiota changes can lead to other issues like colitis development or metabolic syndrome [210]. The alternation includes an increase in pathogenic bacteria. For example studies show an alternation in lingual microbiome composition in HIV infected people that demonstrate an increase in extent of potentially pathogenic Veillonella, Prevotella, Megasphaera, and Campylobacter species [211]. A different study showed an enhancement in Brachyspira,, Catenibacterium, Escherichia, unclassified Enterobacteriaceae, unclassified Fusobacteriaceae, Mogibacterium, and Ralstoniain in HIV patient’s gut microbiota [212]. The oral microbiome can change too, including fungi alternation, as evidence on raising in level of candida, that is Simultaneous, with decreasing in the level of Pichia. Because of pichia inhabitation role against candida via some competitions, like nutrient competition [213]. Also in oropharyngeal microbiome of HIV positive patients, the most common microorganisms are genera Proteus, Enterococcus, Bacteroides, Prevotella and Clostridium [214]. In SIV infection,n such alternations happen too. For example, in chronic SIV infected chimpanzees, a temporary microbiota change was observed [210], or a reduction in Proteobacteria/Succinivibrio was demonstrated in intestinal microbiome of chronic SIV infected vervet monkeys [215].

#### Influenza

Influenza causes an acute infection, which leads to some changes in both intestinal and respiratory microbiome integrity [179]. For example, intense infection with such species of influenza A viruses (IAV), like H1N1 and H5N1 IAV. The microbiota imbalance may enhances the risk of post infection problems, like bacterial superinfection [180]. In influenza infection, the produced type I interferons (IFN-Is) in lungs, cause a reduction in essential anaerobic bacteria and the Proteobacteria enrichment in the gastrointestinal tract, which leads to a dysbiosis in microbiome; also this IFN causes depletion in immune responses against Salmonella-induced colitis and induce Salmonella colonization in gut tract [182]. Additionally, in a case of Salmonella typhimurium superinfection, evidence shows increased sensitivity to bacterial pathogens as a result of intestinal microbiota imbalance [179]. Other study on Chickens infected by Avian Influenza Virus Subtype H9N, showed an elevation in Escherichia species, especially *E.coli*, in addition to a decrease in *Lactobacillus* and *Enterococcus* [216]. The nasopharyngeal (NP) microbiota can change by IAV infection in children. The organisms such as *Moraxella, Staphylococcus, Corynebacterium*, and *Dolosigranulum* have lower plenty in infected NP microbiome community; and usually the *Streptococcus* and the *Phyllobacterium* can be discovered in this site. It seems that in these patients, there are a decrease in *Streptococcus, Neisseria*, and *Haemophilus abundance*. *Cinetobacter*, unclassified Acidobacteria, Ralstonia, Pseudomonas, Lachnoclostridium, and Halomonas also increase notably in patients group. About *oropharengeal*(OP) microbiota, there is some changes too; like depletion in Streptococcus, Neisseria, Haemophilus, Rothia, Fusobacterium, *Granulicatella* and *Gemella abundance* [217]. Other study demonstrated that following species are most detected in upper respiratory tract of influenza A (H1N1) infected patients: *Enterobacteriaceae, Moraxellaceae, Streptococcaceae, Carnobacteriaceae, Pasteurellaceae*, and *Oxalobacteraceae* [218].

#### Norovirus

Human Norovirus (HNoV) infects gastrointestinal tract and causes several acute gastroenteritis as endemic or epidemic forms [219]. HnoV can directly affect gut microbiota by the interaction between its capsid and some bacterial surface carbohydrate as bacterial antigens; another mechanism is binding to sialic acid residues on bacteria and then affecting intestinal microbiota [220]. Murine noroviruses (MNV) causes a disturbance in epithelial barrier in mice intestine, that leads to an imbalances in microbiota community and the hemostasis, which exist in whole immune system of gut tract. This imbalance promotes the disease in patients with inflammatory bowel disease (IBD) risk factors [157]. A study about intestinal microbiota comparison between 2 group of mice (infected and uninfected), no important difference was found; also it showed a resistance in wild-type mice against microbiota alteration via MNV [221]. In both of MNV and HnoV associated microbiota imbalance, an enhance in abundance of *Firmicutes* to *Bacteroidetes* levels is seen [220]. One example of norovirus associated gut microbiome alteration is a reduction in Bacteroidetes and an elevation in Proteobacteria community [222].

#### Rotavirus

Rotavirus (RV) is one of the main causes of gastroenteritis [223] in infants and young age individual, which can lead to a range of problems, from diarrhea to even death. RV infection affects gut microbiota composition. In a study on piglets, elevated ratio of Bacteroidetes and Bacteroidia and decreased ratio of *Firmicutes* and *Erysipelotrichia*, has been found in RV infected piglet’s gut microbiota. Also, remarkable changes are seen in some bacterial groups, which are enrichment of Bacteroidaceae and Helicobacteraceae and depletion in *Porphyromonadaceae, Lactobacillaceae, Erysipelotrichaceae* infected piglets [224]. There is oral form of rotavirus vaccination. Evidence showed that in infants who responded to vaccine, higher level of *Proteobacteria* and *Eggerthella* in gut microbiome was noted, and infants who didn’t respond the vaccine, had higher level of *Fusobacteria* and *Enterobacteriaceae *[225]. Hence, gut microbiota can differently and oppositely affect RV virus.

#### Hepatitis (HBV and HCV)

Hepatitis B virus or hepadnaviruse infects the liver. The infection can be transient or chronic [226]. The chronic infection can lead to cirrhosis, or hepatocellular carcinoma (HCC) [227]. The liver is connected to intestine by 2 way, bile ducts and blood. all the blood away from intestine should pass the liver, so liver diseases can affect intestine and alter the gut microbiota as a side effect of disease [228]. There are some differences in oral microbiota community in hepatitis B virus [HBV) infected person. Evidence show decrease in Firmicutes/Bacteroidetes plenty in patients with chronic liver disease caused by HBV (HBV-CLD). The pattern of oral microbiota alteration which is caused by HBV is same in liver cirrhosis (LC) condition and chronic hepatitis B (CHB). Phylotypes, which produce H2S- and CH3SH, will increase in HBV infection and lead to oral stench. *Fusobacterium, Filifactor, Eubacterium, Parvimonas* and *Treponema* are some of those phylotypes. They can transfer to gut and make an alteration in gut microbiota composition, and the product of this alteration can effect on HBV induced liver disease pathogenesis [229]. Gut dysbiosis, can induce the hepatitis infection development to end stage situation. Viral hepatitis (HBV and HCV) is the most common reason of cirrhosis. Cirrhosis influences the mucosal immune system and make some changes in microbiota in all relative sites. These changes can be seen in fecal microbiota too, like what evidence showed in cirrhotic patient, a depletion in Bacteroidetes ratio and an elevation in Proteobacteria and Fusobacteria was demonstrated in fecal microbiota of these patient. Also, there are some relationships between cirrhosis severity and some bacterial corporator of gut microbiota, for instance, the Streptococcaceae families have a positive relation and the Lachnospiraceae have a negative relation with cirrhosis severity. Another study discovered a relation between fungal variety and bacterial variety in cirrhosis [230]. HCV causes two forms of infection; acute form and chronic form. The patients with chronic hepatitis C are disposed to sever conditions like cirrhosis or hepatocellular carcinoma (HCC). Gut microbiota is altered in patients with chronic Hepatitis C (CHC), such as an elevation in Proteobacteria ratio and a decrease in Bacteroidetes and Firmicutes phyla communities in CHC, HCC and cirrhotic patients. Therefore, it may be indicative of a relationship between microbiota alteration pathogenic effect and the severity of patient’s conditions, like HCC progression. A study showed that the alpha variety of HCV patients gut microbiota is lower than that of healthy patients. The actinobacteria has a higher level and the Bifidobacterium composition just is detected in healthy individuals [231].

### Corona viruses (SARS and COVID-19)

Coronaviruses are enveloped, positive single-stranded RNA viruses, which can infect both human and animals. Seven subtypes of coronaviruses can cause infection in human, which mainly cause common cold. But recently, every decade, newest coronaviruses from beta-coronaviruses such as severe acute respiratory coronavirus (SARS), Middle East Respiratory Syndrome (MERS) and COVID-19 cause global serious health problems [233]. Both SARS and COVID-19, in addition to pneumonia [234], cause a spectrum of gastrointestinal symptoms too [235]. Hence, as discussed in the current study, coronaviruses and microbiota (especially lung microbiota), have mutual interactions as well. It means that they can both regulate the susceptibility to viral infevtons and can be regulated by viral pathogens [236]. And even some of the mechanisms are exactly common with the other viral infections. Further studies may reveal more similarities, more common mechanism and even more new mechanism, which any of them, can become a possible treatment or diagnosis for COVID-19 [237]. For example, HA have general common effects on immune system and viral infection, so is expected to have antiviral effects on COVID-19 [207]. For example, despite the differences between COVID-19 and SARS, they have common receptors like angiotensin-converting enzyme 2 (ACE2). ACE2 has role in some diseases like acute lung injury and also ACE2 is an essential part in gastrointestinal tract functions. ACE2 is required for transporters expression which are related to natural amino acids in gut [238]. Angiotensin-converting enzyme 2 (ACE2) is a common receptor among coronaviruses and is an enzymes in human enterocytes which relates to digestion. Coronaviruses are RNA viruses which have more mutation rather than other families. As a result, during their evolution, changes occur both in their binding receptor and binding modes alternately. But their intestinal target cells unlike those in the lungs, are constant. Studies suggest that both bacterial pathogens and microbiotas or probiotic, elevate probiotics coronaviruses receptors [239]. The amino acid transport function of ACE2 is associated with intestinal microbiota. So that, mutation of ACE2 affects intestinal microbiota composition and lessen antimicrobial peptides expression. In one hand, we have some evidence showing the influence of gut microbiomes on ACE2 actions. For example, In COVID-19, some of these possible intermediations can be done by probiotics species like Bifidobacteria and also Lactobacilli, namely *L. gasseri* [240]. On the other hand, SARS_CoV_2 infection reduces the ACE2, which express in gut tract. Subsequently, circulating angiogenic cells (CACs) will decrease too, and endangers the endothelium, which may leads to intestinal dysbiosis [241]. These evidence indicates the mutual interactions between microbiota and COVID-19 via ACE2 intermediating.

Although it is still not proven by clinical evidence, china’s National Health Commission and National Administration of Traditional Chinese Medicine declared that probiotic administration is beneficial to maintain intestinal microbial balance and preventing secondary/co-infections which can reduce infection mortality. Generally, balanced gut microbiota can reduce enteritis and ventilator-associated pneumonia, and inhibits some viral replications in the lungs such as influenza infection. The lung microbiota can aggravate the infection via changing several factors such as local or systemic inflammatory response, host immunity response, mucosal layers defense and finally exerting secondary bacterial infection [236, 242].

A study conducted by Liu et al., looked at 72 chemical drugs and 27 antibodies, which have key antiviral roles in at least one human coronavirus infection, whether in vitro or in vivo. Many of these drugs inhibit viral entry to cells and viral replication inside cells, or modulate host immune responses. In addition, antimicrobial drugs like antimalarial drugs (e.g. chloroquine and mefloquine), antifungal drugs (e.g. terconazole and rapamycin) and antibiotics (e.g. teicoplanin and azithromycin) are seem to be beneficial in combating against coronaviruses. These antibiotic treatments may have direct effects on infection. or like the examples noted in pervious viruses, may induce changes in microbiota. As the microbiota play a significant role in human metabolism and immune response to viral infections, they can modulate microbiota-mediated mechanisms against or in favor of coronavirus. But it should be mentioned that although they can be used as a preventive method to protect the body against current viral infection or bacterial endogenous or exogenous co-infections, the antibiotic administration may have harmful effects via inducing dysbiosis [243].

Recently Georg Anderson suggests that stress is a factor, which can increase COVID-19 duration and severity via affecting both melatonin and the microbial butyrate. The stress can induces changes in the intestinal microbiota and the gut permeability, and also it can have influence on the circadian rhythm, which both play important roles in immune cell function, especially in response to COVID-19 [244]. Intestinal microbial dysbiosis due to alcohol, sugar and fat full dietary and stress, leads to reduced microbial short-chain fatty acid production, like butyrate, which can enhance immune cells and CNS glia cells operation to suppress COVID-19 infection. The microbiota resales different types of butyrate with different spectrum of antiviral effects. These antiviral activities are due to butyrate epigenetic regulatory properties to block HDAC, which is a common regulator of viruses like the influenza virus, and seems to be beneficial in combating against COVID-19. In addition, butyrate roles in immune responses may be through regulating the melatonergic pathway, which leads to allowing immune cells to turn from an inflammatory to quiescent condition [245, 246].

Regarding intestinal permeability, it should be noted that a leaky gut exports it’s microbiota to the lungs and leads to change pulmonary microbiota. This intestinal microbiota, have key role in the patients enhancement and the need of ventilation and the survival. For example, we saw that elevated intestinal permeability caused by microbiota alterations, can leads to leaking LPS and dietary material into the systemic circulation. This aggravates viral replication and transmission in influenza, HBV and HIV via TLR4 activation, which is a pattern-recognizing receptors (*PRR*) for LPS [137]. Additionally, this pathway leads to releasing immunosuppressive cytokines, decreasing immunoregulatory effects of regulatory T cells and dendritic cells. All these changes lead to viral escape from immune responses and promoting viral infection so that the author hypothesized that it will work against COVID-19 too [244]. Furthermore, MMTV utilize LPS binding proteins such as CD14, TLR4, and MD-2 into its viral envelope for next generations, which may also occur for COVID-19 [149].

Another study on later corona viruses, conducted by Meazzi et al., revealed that the most common microbiota were *Firmicutes, Bacteroidetes* and *Actinobacteria*. So, in FCoV-positive cats, *Firmicutes* and *Bacteroidetes* were more or less than that in FCoV-negative cats. Microbiota similarity was noted among 3 of 5 cats with peritonitis. Thus, some differences can be already detectable. They concluded that changes in the intestinal microbiota composition in cats, may leads to Feline Infectious Peritonitis (FIP) aggravation, which is caused by enteric feline coronaviruses (FCoVs) mutation and disturbs the host immune response. The microbiota can alter FCoV genetic polymorphism, which is associated with the viral replication [247]. Hence, it suspected us that it may be useful in COVID-19 treatment.

The microbiota composition in SARS-CoV-2 and COVID-19 patients is similar to Community acquired pneumonia (CAP) patients. The microbial diversity was significantly lower than normal, with either pathogens or increased oral and upper respiratory microbiota dominancy. But there are no particular microbial patterns in neither of them. In addition, SARS related death is often correlated to acute respiratory distress syndrome (ARDS) [248], and COVID-19 patient’s condition may develop to sever situation like ARDS [249]. ARDS patient usually need mechanical ventilation and it may causes some micro-aspirations. By this way, the gut microbes can transfer to lungs and so lungs microbiome will get enriched by gut bacteria such as *Bacteroidetes* and *Enterobacteriaceae *[250]. Previous studies, showed that the oral and upper respiratory microbiotas in SARS_CoV_2 patients, have elevated levels of pathogens and commensal bacteria [236].

Sometimes microbiota particles such as flagellin act as damage-associated molecular patterns (Damp), and stimulate immune pathways like toll-like receptor 5 (TLR5) pathway. This already have seen in influenza and rotavirus infection. Flagellin is a structural protein in both gram positive and negative bacteria flagellum. It mainly increases anti-bacterial responses but also have anti-viral activities. In Rota virus flagellin activated TLR5 pathway impacts on dendritic cells and led to releasing (IL)-22. Also, it increased NLR Family CARD Domain Containing 4 (NLRC4) dependent IL-18 discharge. IL-22 maintains the epithelial cell and its proliferation normally, while IL-18 eliminates infected epithelial cells via apoptosis [251]. So this pathway immediately removes rotavirus(RV) infection and accelerates RV clearance [252]. It is important to note that flagellin responses are independent of interferon (IFN) responses. Considering that coronaviruses are capable of hijacking type I IFN anti-viral responses through structural and non-structural proteins, utilizing the flagellin-TLR5 axis could provide an effective loophole to target and eliminate SARS-CoV-2. Ironically, it was recently found that flagellin is also capable of inducing TLR5-mediated production of IFN-β and subsequent activation of type I IFN responses, which presents a potential avenue to restore anti-viral immune defenses that are impaired during coronavirus infections [253, 254].

The beneficial role of flagellin viral infections have been proven by several studies. For example, an study revealed therapeutic role of flagellin administration, which can decrease viral load in the lungs of an influenza A virus infected mice [200]. Accompaniment of flagellin with influenza vaccine or inactivated influenza viruses, can leads to higher IgA and IgG titers against influenza via TLR5. These investigations indicate the reinforcing role of flagellin as a therapeutic component and a potent mucosal adjuvant [201]. Moreover, Equine herpesvirus 1 (EHV-1) is a virus from *herpesviridae* family, which induces several distinct pathogeneses, like respiratory infections, viral abortion, neurological signs, and neonatal mortality in horses. This study showed that flagellin has a reinforcing role in HSV vaccination [118]. To conclude the matter, flagellin have significant roles in lung dendritic cells maturation, inhibition of epithelial apoptosis, maintaining epithelial cells and induction of cathelicidin-dependent antimicrobial responses, neonatal lung antigen-presenting cells activation [253, 255]. All of these are important in COVID-19 infection and suggest the beneficial roles of microbiota derived flagellin in this infection.

In the other hand, some studies reported that flagellin TLR5 activation of NF-κB signaling, promotes lentiviral pseudovirus attachment on lung epithelial cells. So TLR5 activation may promote SARS-CoV-2 infection and induces harmful inflammatory responses. So just IL-18 and IL-22 administration would have the beneficial effects within harmful effects. In any case, the lack of immune responses exhibited in the early stage of coronavirus infections, may suggest that TLR5 agonists could be most valuable in the early phase of infection, whereas the media hype chloroquine could be most useful against late stage infection, since chloroquine is an inhibitor of nucleic acid recognizing TLR-mediated inflammatory responses [253]. Regardless, the benefits or costs of TLR5 activation would still expand the mechanistic knowledge on COVID-19 pathogenesis and direct us toward appropriate therapeutic targets at the pertinent viral stage. In line with TLR5, degradation of NETs through 162 host nucleases might have potential risks, as such intervention has been reported as a fuel source for certain pathogens, such as Haemophilus influenzae [11]. Hence, if SARS CoV-2 utilizes a similar strategy, this could also advance its pathogenesis. Yet, there is no article to date that has associated coronaviruses to utilize NETs degradation via host nucleases for virulence; therefore, studying this relationship could provide one of two novel findings: (i) an unexplored, yet new, mechanism for coronavirus virulence or (ii) a potential therapeutic (i. E. DNase I) to defeat COVID-19 through regulation of aberrant innate immune responses [253]. Based on our previous studies of flagellin protection against rotavirus infection, we hypothesized that flagellin would be most potent within the first 48 h of infection, as this will allow for early intervention to boost anti-viral responses and block virulence.

## Conclusions

At the end we have to mention again, although there are articles indicating the role of microbiota in viral infection and the viruses on microbiota composition, our understanding on how do they work, is not enough. Moreover, the issue does not involve just a mutual interaction between single pair of microorganism species, but we face a complex network of pathways between different hosts different microbiotas and different viruses. So that previous reports demonstrated that bacterial flagellin promotes viral infections like influenza, Measles, Ebola, Lassa, and Vesicular stomatitis virus in pulmonary epithelial cell culture through TLR5 and NF-κB activation [256], although previously flagellin was known by its inhibitory effects against RV infection in mice [142]. These paradoxical results are result of variances in the microenvironment and models utilized in these studies. We remind them to demonstrate how much these interactions are complex and unknown. However, knowing about these mechanisms between the microbiota and viral infections, helps to produce both chemical medications originated from bacterial secretions or manufactured via bioengineering, and probiotic medications, which can colonize in the body and induce their related antiviral responses. In addition, they can be utilized as a booster for pervious treatments and vaccine immunology. In the field of vaccinology, we found the potent immunogenic role of microbiotas as oral enteric vaccines, and specially rotavirus vaccine, to improve vaccines efficacy. Also data confirms that the microbiota targets would stay related to both specific vaccines and patient populations [232]. It is noteworthy to mention that although we mainly recall viruses as pathogens, but there are some viruses called as virome, which are commensal microbiota in healthy peoples and have been remain understudied. They include bacteriophages and plants microbiota and also eukaryotic viruses, which their existence in every individual, depends on the host commensal bacteria and diet. They might also have some impacts on viral infection either in a suppressive or aggregative fashion, but they need to be studied more. Finally, microbiome biobanks is needed to be expanded for both human or animals., which can help in future studies on biomedical research, and industry [257].

## Data Availability

Not applicable.

## References

[1] Rowan-Nash AD, Korry BJ, Mylonakis E, Belenky P (2019). Cross-domain and viral interactions in the microbiome. Microbiol Mol Biol Rev.

[2] Ahern PP, Maloy KJ (2020). Understanding immune–microbiota interactions in the intestine. Immunology.

[3] Proctor DM, Relman DA (2017). The landscape ecology and microbiota of the human nose, mouth, and throat. Cell Host Microbe.

[4] Stapleton AE. The vaginal microbiota and urinary tract infection. Urin Tract Infect. 2017:79–86. 10.1128/microbiolspec.UTI-0025-2016 PMC5746606 28087949

[5] Chen YE, Fischbach MA, Belkaid Y (2018). Skin microbiota–host interactions. Nature.

[6] Pfeiffer JK, Sonnenburg JL (2011). The intestinal microbiota and viral susceptibility. Front Microbiol.

[7] Karst SM (2016). The influence of commensal bacteria on infection with enteric viruses. Nat Rev Microbiol.

[8] Khoruts A (2016). First microbial encounters. Nature medicine.

[9] Nuriel-Ohayon M, Neuman H, Koren O (2016). Microbial changes during pregnancy, birth, and infancy. Frontiers in microbiology.

[10] Dominguez-Bello MG, Costello EK, Contreras M, Magris M, Hidalgo G, Fierer N (2010). Delivery mode shapes the acquisition and structure of the initial microbiota across multiple body habitats in newborns. Proc Natl Acad Sci..

[11] Rautava S (2016). Early microbial contact, the breast milk microbiome and child health. J Dev Orig Health Dis.

[12] Garber A, Hastie P, Murray J-A (2020). Factors influencing equine gut microbiota: current knowledge. J Equine Vet Sci..

[13] Liu J, Lahousse L, Nivard MG, Bot M, Chen L, van Klinken JB (2020). Integration of epidemiologic, pharmacologic, genetic and gut microbiome data in a drug–metabolite atlas. Nat Med.

[14] Goodrich JK, Davenport ER, Beaumont M, Jackson MA, Knight R, Ober C (2016). Genetic determinants of the gut microbiome in UK twins. Cell Host Microbe.

[15] Gomez A, Espinoza JL, Harkins DM, Leong P, Saffery R, Bockmann M (2017). Host genetic control of the oral microbiome in health and disease. Cell Host Microbe.

[16] Fan P, Bian B, Teng L, Nelson CD, Driver J, Elzo MA (2020). Host genetic effects upon the early gut microbiota in a bovine model with graduated spectrum of genetic variation. The ISME Journal.

[17] Rothschild D, Weissbrod O, Barkan E, Kurilshikov A, Korem T, Zeevi D (2018). Environment dominates over host genetics in shaping human gut microbiota. Nature.

[18] Moreno-Gallego JL, Chou S-P, Di Rienzi SC, Goodrich JK, Spector TD, Bell JT (2019). Virome diversity correlates with intestinal microbiome diversity in adult monozygotic twins. Cell Host Microbe.

[19] Stubbendieck RM, May DS, Chevrette MG, Temkin MI, Wendt-Pienkowski E, Cagnazzo J (2019). Competition among nasal bacteria suggests a role for siderophore-mediated interactions in shaping the human nasal microbiota. Appl Environ Microbiol.

[20] Albenberg L, Esipova TV, Judge CP, Bittinger K, Chen J, Laughlin A (2014). Correlation between intraluminal oxygen gradient and radial partitioning of intestinal microbiota. Gastroenterology.

[21] Hord NG (2008). Eukaryotic-microbiota crosstalk: potential mechanisms for health benefits of prebiotics and probiotics. Annu Rev Nutr.

[22] Malaguarnera L (2020). Vitamin D and microbiota: Two sides of the same coin in the immunomodulatory aspects. Int Immunopharmacol.

[23] Keyel PA (2014). How is inflammation initiated? Individual influences of IL-1, IL-18 and HMGB1. Cytokine.

[24] Lazar V, Ditu L-M, Pircalabioru GG, Gheorghe I, Curutiu C, Holban AM (2018). Aspects of gut microbiota and immune system interactions in infectious diseases, immunopathology, and cancer. Frontiers in immunology.

[25] Ubeda C, Pamer EG (2012). Antibiotics, microbiota, and immune defense. Trends Immunol.

[26] Cianci R, Pagliari D, Piccirillo CA, Fritz JH, Gambassi G (2018). The microbiota and immune system crosstalk in health and disease. Mediat Inflamm.

[27] Stecher B, Hardt W-D (2008). The role of microbiota in infectious disease. Trends in microbiology.

[28] Forbes JD, Van Domselaar G, Bernstein CN (2016). The gut microbiota in immune-mediated inflammatory diseases. Frontiers in microbiology.

[29] Kamada N, Seo S-U, Chen GY, Núñez G (2013). Role of the gut microbiota in immunity and inflammatory disease. Nat Rev Immunol.

[30] Raskov H, Bjarnsholt T, Alamili M, Kragh K, Gögenur I (2018). Interaction between microbiota and immune system in colorectal cancer. Ugeskrift for laeger.

[31] Meurman JH (2010). Oral microbiota and cancer. Journal of oral microbiology.

[32] Wu H-J, Wu E (2012). The role of gut microbiota in immune homeostasis and autoimmunity. Gut microbes.

[33] Sokolowska M, Frei R, Lunjani N, Akdis CA, O’Mahony L (2018). Microbiome and asthma. Asthma research practice.

[34] Jama HA, Kaye DM, Marques FZ (2019). The gut microbiota and blood pressure in experimental models. Curr Opin Nephrol Hypertens.

[35] Tang WW, Kitai T, Hazen SL (2017). Gut microbiota in cardiovascular health and disease. Circulat Res.

[36] Kazemian N, Mahmoudi M, Halperin F, Wu JC, Pakpour S (2020). Gut microbiota and cardiovascular disease: opportunities and challenges. Microbiome.

[37] Karpiński TM (2019). Role of oral microbiota in cancer development. Microorganisms.

[38] Garrett WS (2015). Cancer and the microbiota. Science.

[39] Yu G, Gail MH, Shi J, Klepac-Ceraj V, Paster BJ, Dye BA (2014). Association between upper digestive tract microbiota and cancer-predisposing states in the esophagus and stomach. Cancer Epidemiol Prev Biomarkers.

[40] Shillitoe EJ (2018). The Microbiome of Oral Cancer. Critical Reviews™ in Oncogenesis.

[41] Maisonneuve C, Irrazabal T, Martin A, Girardin SE, Philpott DJ. The impact of the gut microbiome on colorectal cancer. 2018.

[42] Mao Q, Jiang F, Yin R, Wang J, Xia W, Dong G (2018). Interplay between the lung microbiome and lung cancer. Cancer Lett.

[43] Perez-Chanona E, Trinchieri G (2016). The role of microbiota in cancer therapy. Curr Opin Immunol.

[44] Vivarelli S, Salemi R, Candido S, Falzone L, Santagati M, Stefani S (2019). Gut microbiota and cancer: From pathogenesis to therapy. Cancers.

[45] Nagao-Kitamoto H, Kitamoto S, Kuffa P, Kamada N (2016). Pathogenic role of the gut microbiota in gastrointestinal diseases. Intestinal research.

[46] Rowland I, Gibson G, Heinken A, Scott K, Swann J, Thiele I (2018). Gut microbiota functions: metabolism of nutrients and other food components. Eur J Nutr.

[47] Thaiss CA, Levy M, Korem T, Dohnalová L, Shapiro H, Jaitin DA (2016). Microbiota diurnal rhythmicity programs host transcriptome oscillations. Cell.

[48] Tilg H, Adolph TE, Gerner RR, Moschen AR (2018). The intestinal microbiota in colorectal cancer. Cancer Cell.

[49] Gao R, Gao Z, Huang L, Qin H (2017). Gut microbiota and colorectal cancer. European Journal of Clinical Microbiology Infectious Diseases.

[50] Wang S, Blachier F, Zhao F, Yin Y (2011). Intestinal microbiota: Development, metabolism and functions. J Food Agric Environ.

[51] Leong KS, Derraik JG, Hofman PL, Cutfield WS (2018). Antibiotics, gut microbiome and obesity. Clin Endocrinol.

[52] Cani PD. 16 Gut Microbiome and Obesity. Handbook of Obesity, Two-Volume Set. 2019:183.

[53] Martin AM, Sun EW, Rogers GB, Keating DJ (2019). The influence of the gut microbiome on host metabolism through the regulation of gut hormone release. Front Physiol.

[54] Davis-Richardson AG, Ardissone AN, Dias R, Simell V, Leonard MT, Kemppainen KM (2014). Bacteroides dorei dominates gut microbiome prior to autoimmunity in Finnish children at high risk for type 1 diabetes. Front Microbiol.

[55] Zheng P, Li Z, Zhou Z (2018). Gut microbiome in type 1 diabetes: a comprehensive review. Diab/Metab Res Rev.

[56] Hidalgo-Cantabrana C, Delgado S, Ruiz L, Ruas-Madiedo P, Sánchez B, Margolles A. Bifidobacteria and their health-promoting effects. Bugs as Drugs: Therapeutic Microbes for the Prevention and Treatment of Disease. 2018:73–98.

[57] Bibbò S, Dore MP, Pes GM, Delitala G, Delitala AP (2017). Is there a role for gut microbiota in type 1 diabetes pathogenesis?. Ann Med.

[58] Tai N, Peng J, Liu F, Gulden E, Hu Y, Zhang X (2016). Microbial antigen mimics activate diabetogenic CD8 T cells in NOD mice. J Exp Med.

[59] Hu Y, Wong FS, Wen L (2017). Antibiotics, gut microbiota, environment in early life and type 1 diabetes. Pharmacol Res.

[60] Long J, Cai Q, Steinwandel M, Hargreaves MK, Bordenstein SR, Blot WJ (2017). Association of oral microbiome with type 2 diabetes risk. J Periodontal Res.

[61] Obata Y, Pachnis V (2016). The effect of microbiota and the immune system on the development and organization of the enteric nervous system. Gastroenterology.

[62] Jiang C, Li G, Huang P, Liu Z, Zhao B (2017). The gut microbiota and Alzheimer’s disease. J Alzheimers Dis.

[63] Ghomi R, Nemani K (2016). The influence of diet and the gut microbiota in Schizophrenia.&nbsp;The gut-brain axis.

[64] Berer K, Gerdes LA, Cekanaviciute E, Jia X, Xiao L, Xia Z (2017). Gut microbiota from multiple sclerosis patients enables spontaneous autoimmune encephalomyelitis in mice. Proc Natl Acad Sci..

[65] Philip V. Microbiota induced immune system maturation plays a key role in development of normal behaviour 2017.

[66] Foster JA, Neufeld K-AM (2013). Gut–brain axis: how the microbiome influences anxiety and depression. Trends&nbsp; Neurosci.

[67] Kabouridis PS, Pachnis V (2015). Emerging roles of gut microbiota and the immune system in the development of the enteric nervous system. J Clin Investig.

[68] De Vadder F, Grasset E, Holm LM, Karsenty G, Macpherson AJ, Olofsson LE (2018). Gut microbiota regulates maturation of the adult enteric nervous system via enteric serotonin networks. Proc Natll Acad Sci..

[69] Toral Jiménez M, Robles-Vera I, de la Visitación N, Romero M, Yang T, Sánchez M, et al. Critical Role of the Interaction Gut Microbiota–Sympathetic Nervous System in the Regulation of Blood Pressure. 2019. 10.3389/fphys.2019.00231 PMC6423906 30930793

[70] Wypych TP, Wickramasinghe LC, Marsland BJ (2019). The influence of the microbiome on respiratory health. Nat Immunol.

[71] Unger SA, Bogaert D (2017). The respiratory microbiome and respiratory infections. J Infect.

[72] Boutin S, Graeber SY, Stahl M, Dittrich AS, Mall MA, Dalpke AH (2017). Chronic but not intermittent infection with Pseudomonas aeruginosa is associated with global changes of the lung microbiome in cystic fibrosis. Eur Respir J.

[73] Hewitt RJ, Molyneaux PL (2017). The respiratory microbiome in idiopathic pulmonary fibrosis. Ann Transl Med..

[74] Xunliang T, Fei S, Xiaomao X, Hongtao X, Ting Y, Qixia X (2019). Alterations to the lung microbiome in idiopathic pulmonary fibrosis patients. Front Cell Infect Microbiol.

[75] Richardson H, Dicker AJ, Barclay H, Chalmers JD (2019). The microbiome in bronchiectasis. Eur Respir Rev.

[76] Zhang D, Li S, Wang N, Tan H-Y, Zhang Z, Feng Y (2020). The cross-talk between gut microbiota and lungs in common lung diseases. Front Microbiol..

[77] Chun Y, Do A, Grishina G, Grishin A, Fang G, Rose S (2020). Integrative study of the upper and lower airway microbiome and transcriptome in asthma. JCI Insight..

[78] Voronina O, Ryzhova N, Kunda M, Loseva E, Aksenova E, Amelina E (2020). Characteristics of the airway microbiome of cystic fibrosis patients. Biochemistry.

[79] Pu CY, Seshadri M, Manuballa S, Yendamuri S (2020). The oral microbiome and lung diseases. Curr Oral Health Rep.

[80] Wenfang H, Yueyun M, Zhou L, Hao X (2016). The role of microbiome in respiratory disease. Chin J Lab Med.

[81] Adar SD, Huffnagle GB, Curtis JL (2016). The respiratory microbiome: an underappreciated player in the human response to inhaled pollutants?. Ann Epidemiol.

[82] Buffie CG, Pamer EG (2013). Microbiota-mediated colonization resistance against intestinal pathogens. Nat Rev Immunol.

[83] Chang PV (2020). Chemical mechanisms of colonization resistance by the gut microbial metabolome. ACS Chem Biol..

[84] Ubeda C, Djukovic A, Isaac S (2017). Roles of the intestinal microbiota in pathogen protection. Clin Transl Immunol.

[85] Umu ÖC, Rudi K, Diep DB (2017). Modulation of the gut microbiota by prebiotic fibres and bacteriocins. Microbial ecology in health disease.

[86] Wilks J, Beilinson H, Golovkina TV (2013). Dual role of commensal bacteria in viral infections. Immunol Rev.

[87] Vogt SL, Finlay BB (2017). Gut microbiota-mediated protection against diarrheal infections. J Travel Med.

[88] O’Sullivan JN, Rea MC, O’Connor PM, Hill C, Ross RP (2019). Human skin microbiota is a rich source of bacteriocin-producing staphylococci that kill human pathogens. FEMS Microbiol Ecol.

[89] Lima MT, Andrade ACSP, Oliveira GP, Nicoli JR, dos Santos Martins F, Kroon EG (2019). Virus and microbiota relationships in humans and other mammals: An evolutionary view. Human Microbiome J..

[90] Wilks J, Golovkina T (2012). Influence of microbiota on viral infections. PLoS Pathog..

[91] Ma W-T, Pang M, Fan Q-L, Chen D-K (2019). The commensal microbiota and viral infection: a comprehensive review. Front Immunol.

[92] Kennedy EA, King KY, Baldridge MT (2018). Mouse microbiota models: comparing germ-free mice and antibiotics treatment as tools for modifying gut bacteria. Front Physiol.

[93] Nácher-Vázquez M, Ballesteros N, Canales Á, Saint-Jean SR, Pérez-Prieto SI, Prieto A (2015). Dextrans produced by lactic acid bacteria exhibit antiviral and immunomodulatory activity against salmonid viruses. Carbohydr Polym.

[94] Botić T, Danø T, Weingartl H, Cencič A (2007). A novel eukaryotic cell culture model to study antiviral activity of potential probiotic bacteria. Int J Food Microbiol.

[95] Martín V, Maldonado A, Fernández L, Rodríguez JM, Connor RI (2010). Inhibition of human immunodeficiency virus type 1 by lactic acid bacteria from human breastmilk. Breastfeed Med.

[96] Kwasniewski W, Wolun–Cholewa M, Kotarski J, Warchol W, Kuzma D, Kwasniewska A (2018). Microbiota dysbiosis is associated with HPV–induced cervical carcinogenesis. Oncol Lett.

[97] Petrova MI, Lievens E, Malik S, Imholz N, Lebeer S (2015). Lactobacillus species as biomarkers and agents that can promote various aspects of vaginal health. Front Physiol.

[98] Nardis C, Mosca L, Mastromarino P (2013). Vaginal microbiota and viral sexually transmitted diseases. Ann Ig.

[99] Manuel VHV (2018). Vaginal Microbiota and Bacterial Vaginosis. Online J Gyne Obste Maternity Care.

[100] Bolton M, Van Der Straten A, Cohen CR (2008). Probiotics: potential to prevent HIV and sexually transmitted infections in women. Sex Transm Dis.

[101] Petrova MI, van den Broek M, Balzarini J, Vanderleyden J, Lebeer S (2013). Vaginal microbiota and its role in HIV transmission and infection. FEMS MicroBiol Rev.

[102] Malik S. Molecular Study of Mannose Binding Lectin (s) of Lactobacilli and their Potential as HIV Trap. 2014.

[103] Conti C, Malacrino C, Mastromarino P (2009). Inhibition of herpes simplex virus type 2 by vaginal lactobacilli. J Physiol Pharmacol.

[104] Mirmonsef P, Krass L, Landay A, Spear T (2012). The role of bacterial vaginosis and trichomonas in HIV transmission across the female genital tract. Curr HIV Res.

[105] Balkus JE, Mitchell C, Agnew K, Liu C, Fiedler T, Cohn SE (2012). Detection of hydrogen peroxide-producing Lactobacillus species in the vagina: a comparison of culture and quantitative PCR among HIV-1 seropositive women. BMC Infect Dis.

[106] Schellenberg JJ, Plummer FA (2012). The microbiological context of HIV resistance: vaginal microbiota and mucosal inflammation at the viral point of entry. Int J Inflamm.

[107] Keller MJ, Huber A, Espinoza L, Serrano MG, Parikh HI, Buck GA (2019). Impact of Herpes Simplex Virus Type 2 and Human Immunodeficiency Virus dual infection on female genital tract mucosal immunity and the vaginal microbiome. J Infect Dis.

[108] Drider D, Bendali F, Naghmouchi K, Chikindas ML (2016). Bacteriocins: not only antibacterial agents. Probiot Antimicrob Proteins.

[109] Férir G, Petrova MI, Andrei G, Huskens D, Hoorelbeke B, Snoeck R (2013). Labyrinthopeptin A1, a novel lantibiotic peptide, is a dual HIV and HSV entry inhibitor. Rev Antiviral Ther.

[110] Thaiss CA, Zmora N, Levy M, Elinav E (2016). The microbiome and innate immunity. Nature.

[111] Abt MC, Osborne LC, Monticelli LA, Doering TA, Alenghat T, Sonnenberg GF (2012). Commensal bacteria calibrate the activation threshold of innate antiviral immunity. Immunity.

[112] Hernández PP, Mahlakõiv T, Yang I, Schwierzeck V, Nguyen N, Guendel F (2015). Interferon-λ and interleukin 22 act synergistically for the induction of interferon-stimulated genes and control of rotavirus infection. Nat Immunol.

[113] Ichinohe T, Pang IK, Kumamoto Y, Peaper DR, Ho JH, Murray TS (2011). Microbiota regulates immune defense against respiratory tract influenza A virus infection. Proc Natl Acad Sci..

[114] Whitlow A, Herndon MK, Bova J, Campbell R (2019). Interactions between genital microbiota and viral sexually transmitted infections: transmission, prevention, and treatment. Curr Clin Microbiol Rep.

[115] Mousavi E, Makvandi M, Teimoori A, Ataei A, Ghafari S, Samarbaf-Zadeh A (2018). Antiviral effects of *Lactobacillus crispatus *against HSV-2 in mammalian cell lines. J Chin Med Assoc.

[116] Mastromarino P, Cacciotti F, Masci A, Mosca L (2011). Antiviral activity of Lactobacillus brevis towards herpes simplex virus type 2: role of cell wall associated components. Anaerobe.

[117] Williams B, Landay A, Presti RM (2016). Microbiome alterations in HIV infection a review. Cell Microbiol.

[118] Zhao Y, Chang J, Zhang B, Tong P, Wang C, Ran D (2019). TLR-5 agonist *Salmonella abortus *equi flagellin FliC enhances FliC-gD-based DNA vaccination against equine herpesvirus 1 infection. Arch Virol.

[119] d’Ettorre G, Ceccarelli G, Giustini N, Serafino S, Calantone N, De Girolamo G (2015). Probiotics reduce inflammation in antiretroviral treated, HIV-infected individuals: results of the “Probio-HIV” clinical trial. PLoS ONE..

[120] Hummelen R, Changalucha J, Butamanya NL, Koyama TE, Cook A, Habbema JDF (2011). Effect of 25 weeks probiotic supplementation on immune function of HIV patients. Gut Microbes.

[121] Torcia MG (2019). Interplay among vaginal microbiome, immune response and sexually transmitted viral infections. Int J Mol Sci.

[122] Reikvam DH, Meyer-Myklestad MH, Trøseid M, Stiksrud B (2020). Probiotics to manage inflammation in HIV infection. Curr Opin Infect Dis.

[123] Oh JE, Kim B-C, Chang D-H, Kwon M, Lee SY, Kang D (2016). Dysbiosis-induced IL-33 contributes to impaired antiviral immunity in the genital mucosa. Proc Natl Acad Sci..

[124] Pusch O, Boden D, Hannify S, Lee F, Tucker LD, Boyd MR (2005). Bioengineering lactic acid bacteria to secrete the HIV-1 virucide cyanovirin. JAIDS J Acquir Immune Defic Syndr.

[125] Singh B, Mal G, Gautam SK, Mukesh M (2019). Nutraceuticals from bioengineered microorganisms. Advances in animal biotechnology.

[126] Singh B, Mal G, Marotta F (2017). Designer probiotics: paving the way to living therapeutics. Trends Biotechnol.

[127] Ratajczak W, Rył A, Mizerski A, Walczakiewicz K, Sipak O, Laszczyńska M (2019). Immunomodulatory potential of gut microbiome-derived short-chain fatty acids (SCFAs). Acta Biochim Pol.

[128] Benedikz EK. The effect of bacterial flagellin on virus infection: University of Birmingham; 2017.

[129] Aghamiri S, Talaei S, Roshanzamiri S, Zandsalimi F, Fazeli E, Aliyu M (2020). Delivery of genome editing tools: a promising strategy for HPV-related cervical malignancy therapy. Exp Opin Drug Deliv..

[130] Lee JE, Lee S, Lee H, Song Y-M, Lee K, Han MJ (2013). Association of the vaginal microbiota with human papillomavirus infection in a Korean twin cohort. PloS ONE..

[131] Gao W, Weng J, Gao Y, Chen X (2013). Comparison of the vaginal microbiota diversity of women with and without human papillomavirus infection: a cross-sectional study. BMC Infect Dis.

[132] Castañeda-Avila MA, Suárez-Pérez E, Bernabe-Dones R, Unger ER, Panicker G, Ortiz AP (2020). Chlamydia Trachomatis and Human Papillomavirus Serostatus in Puerto Rican Women. P R Health Sci J.

[133] Lugo LZ, Jacob C, Machado AP, Almeida FG, Ávila LS, Prata T (2018). Human papillomavirus and coinfections with Chlamydia trachomatis, Gardnerella vaginalis, and Trichomonas vaginalis in self-collected samples from female sex workers in the Central-Western region of Brazil. J Brasileiro de Patologia e Medicina Laboratorial.

[134] Piyathilake CJ, Ollberding NJ, Kumar R, Macaluso M, Alvarez RD, Morrow CD (2016). Cervical microbiota associated with higher grade cervical intraepithelial neoplasia in women infected with high-risk human papillomaviruses. Cancer Prev Res.

[135] Audsley MD, Yixin HY, McGraw EA (2017). The microbiome composition of Aedes aegypti is not critical for Wolbachia-mediated inhibition of dengue virus. PLoS Negl Trop Dis.

[136] Xi Z, Ramirez JL, Dimopoulos G (2008). The Aedes aegypti toll pathway controls dengue virus infection. PLoS Pathog..

[137] Robinson CM, Pfeiffer JK (2014). Viruses and the microbiota. Annu Rev Virol.

[138] Domínguez-Díaz C, García-Orozco A, Riera-Leal A, Padilla-Arellano JR, Fafutis-Morris M (2019). Microbiota and its role on viral evasion: Is it with us or against us?. Front Cell Infect Microbiol..

[139] Gravinatti ML, Barbosa CM, Soares RM, Gregori F (2020). Synanthropic rodents as virus reservoirs and transmitters. Rev Soc Bras Med Trop.

[140] Kernbauer E, Ding Y, Cadwell K (2014). An enteric virus can replace the beneficial function of commensal bacteria. Nature.

[141] Zhang M, Zhang M, Zhang C, Du H, Wei G, Pang X (2009). Pattern extraction of structural responses of gut microbiota to rotavirus infection via multivariate statistical analysis of clone library data. FEMS Microbiol Ecol.

[142] Zhang T, Lin W (2014). Metal–organic frameworks for artificial photosynthesis and photocatalysis. Chem Soc Rev.

[143] Rigo-Adrover MdM, Van Limpt K, Knipping K, Garssen J, Knol J, Costabile A (2018). Preventive effect of a synbiotic combination of galacto-and fructooligosaccharides mixture with Bifidobacterium breve M-16 V in a model of multiple rotavirus infections. Front Immunol.

[144] Uchiyama R, Chassaing B, Zhang B, Gewirtz AT (2014). Antibiotic treatment suppresses rotavirus infection and enhances specific humoral immunity. J Infect Dis.

[145] Harris VC, Haak BW, Handley SA, Jiang B, Velasquez DE, Hykes BL (2018). Effect of antibiotic-mediated microbiome modulation on rotavirus vaccine immunogenicity: a human, randomized-control proof-of-concept trial. Cell Host Microbe.

[146] Harris VC, Haak BW, Handley SA, Jiang B, Velasquez DE, Hykes BL, et al. Prospective modulation of the gut microbiome boosts rotavirus vaccine immunogenicity in a randomized controlled trial.

[147] Monedero V, Buesa J, Rodríguez-Díaz J (2018). The interactions between host glycobiology, bacterial microbiota, and viruses in the gut. Viruses.

[148] Cardemil CV, O’Leary ST, Beaty BL, Ivey K, Lindley MC, Kempe A (2020). Primary care physician knowledge, attitudes, and diagnostic testing practices for norovirus and acute gastroenteritis. Plos ONE.

[149] Roth AN, Grau KR, Karst SM (2019). Diverse mechanisms underlie enhancement of enteric viruses by the mammalian intestinal microbiota. Viruses.

[150] Franck KT, Fonager J, Ersbøll AK, Böttiger B (2014). Norovirus epidemiology in community and health care settings and association with patient age. DenEmerg Infect Dis.

[151] Walker FC, Baldridge MT (2019). Interactions between noroviruses, the host, and the microbiota. Curr Opin Virol.

[152] Chhabra P, Ranjan P, Cromeans T, Sambhara S, Vinjé J (2017). Critical role of RIG-I and MDA5 in early and late stages of Tulane virus infection. J Gen Virol.

[153] Lee H, Ko G (2016). Antiviral effect of vitamin A on norovirus infection via modulation of the gut microbiome. Sci Rep.

[154] Lee H, Ko G (2016). Antiviral effect of vitamin A on norovirus infection via modulation of the gut microbiome. Sci Rep.

[155] Li D, Breiman A, Le Pendu J, Uyttendaele M (2015). Binding to histo-blood group antigen-expressing bacteria protects human norovirus from acute heat stress. Front Microbiol.

[156] Jones MK, Watanabe M, Zhu S, Graves CL, Keyes LR, Grau KR (2014). Enteric bacteria promote human and mouse norovirus infection of B cells. Science.

[157] Basic M, Keubler LM, Buettner M, Achard M, Breves G, Schröder B (2014). Norovirus triggered microbiota-driven mucosal inflammation in interleukin 10-deficient mice. Inflamm Bowel Dis.

[158] Santiana M, Ghosh S, Ho BA, Rajasekaran V, Du W-L, Mutsafi Y (2018). Vesicle-cloaked virus clusters are optimal units for inter-organismal viral transmission. Cell Host Microbe.

[159] Berger AK, Yi H, Kearns DB, Mainou BA (2017). Bacteria and bacterial envelope components enhance mammalian reovirus thermostability. PLoS Pathog.

[160] Kuss SK, Best GT, Etheredge CA, Pruijssers AJ, Frierson JM, Hooper LV (2011). Intestinal microbiota promote enteric virus replication and systemic pathogenesis. Science.

[161] Robinson CM, Jesudhasan PR, Pfeiffer JK (2014). Bacterial lipopolysaccharide binding enhances virion stability and promotes environmental fitness of an enteric virus. Cell Host Microbe.

[162] Mazel-Sanchez B, Yildiz S, Schmolke M (2019). Menage a trois: virus, host, and microbiota in experimental infection models. Trends Microbiol.

[163] Yao H, Wang X, Song J, Wang Y, Song Q, Han J (2020). Coxsackievirus B3 infection induces changes in the expression of numerous piRNAs. Arch Virol.

[164] Acevedo MAW, Pfeiffer JK. Microbiota-Independent Antiviral Effects of Antibiotics on Enteric Viruses. BioRxiv. 2020. 10.1016/j.virol.2020.04.001 PMC7253499 32452414

[165] Goddard AF, Staudinger BJ, Dowd SE, Joshi-Datar A, Wolcott RD, Aitken ML (2012). Direct sampling of cystic fibrosis lungs indicates that DNA-based analyses of upper-airway specimens can misrepresent lung microbiota. Proc Natl Acad Sci..

[166] Coburn B, Wang PW, Caballero JD, Clark ST, Brahma V, Donaldson S (2015). Lung microbiota across age and disease stage in cystic fibrosis. Sci Rep.

[167] Hong B-y, Paulson JN, Stine OC, Weinstock GM, Cervantes JL (2018). Meta-analysis of the lung microbiota in pulmonary tuberculosis. Tuberculosis.

[168] Dickson RP, Schultz MJ, van der Poll T, Schouten LR, Falkowski NR, Luth JE (2020). Lung microbiota predict clinical outcomes in critically ill patients. Am J Respir Crit Care Med.

[169] Yang D, Xing Y, Song X, Qian Y (2020). The impact of lung microbiota dysbiosis on inflammation. Immunology.

[170] Hui AW-H, Lau H-W, Chan TH-T, Tsui SK-W (2013). The human microbiota: a new direction in the investigation of thoracic diseases. J Thorac Dis.

[171] Charlson ES, Bittinger K, Haas AR, Fitzgerald AS, Frank I, Yadav A (2011). Topographical continuity of bacterial populations in the healthy human respiratory tract. Am J Respir Crit Care Med.

[172] Lynch SV (2014). Viruses and microbiome alterations. Ann Am Thorac Soc.

[173] Yang Z, Bochkov YA, Voelker DR, Foster MW, Que LG (2019). Identification of a novel inhibitor of human rhinovirus replication and inflammation in airway epithelial cells. Am J Respir Cell Mol Biol.

[174] Lamborn IT, Su HC (2020). Genetic determinants of host immunity against human rhinovirus infections. Hum Gen..

[175] Toivonen L, Camargo CA, Gern JE, Bochkov YA, Mansbach JM, Piedra PA (2019). Association between rhinovirus species and nasopharyngeal microbiota in infants with severe bronchiolitis. J Allergy Clin Immunol.

[176] Korten I, Ramsey K, Mika M, Usemann J, Frey U, Hilty M (2019). Nasal microbiota and respiratory tract infections: the role of viral detection. Am J Respir Crit Care Med.

[177] Tamura S-i, Kurata T (2004). Defense mechanisms against influenza virus infection in the respiratory tract mucosa. Jpn J Infect Dis.

[178] Liu C, Eichelberger MC, Compans RW, Air GM (1995). Influenza type A virus neuraminidase does not play a role in viral entry, replication, assembly, or budding. J Virol.

[179] Yildiz S, Mazel-Sanchez B, Kandasamy M, Manicassamy B, Schmolke M (2018). Influenza A virus infection impacts systemic microbiota dynamics and causes quantitative enteric dysbiosis. Microbiome.

[180] Sencio V, Barthelemy A, Tavares LP, Machado MG, Soulard D, Cuinat C (2020). Gut dysbiosis during influenza contributes to pulmonary Pneumococcal superinfection through altered short-chain fatty acid production. Cell Rep.

[181] Wang J, Li F, Wei H, Lian Z-X, Sun R, Tian Z (2014). Respiratory influenza virus infection induces intestinal immune injury via microbiota-mediated Th17 cell–dependent inflammation. J Exp Med.

[182] Deriu E, Boxx GM, He X, Pan C, Benavidez SD, Cen L (2016). Influenza virus affects intestinal microbiota and secondary salmonella infection in the gut through type I interferons. PLoS Pathog.

[183] Qin N, Zheng B, Yao J, Guo L, Zuo J, Wu L (2015). Influence of H7N9 virus infection and associated treatment on human gut microbiota. Sci Rep.

[184] Figueroa T, Bessière P, Coggon A, Bouwman KM, van der Woude R, Delverdier M (2020). The microbiota contributes to the control of highly pathogenic H5N9 influenza virus replication in Ducks. J Virol..

[185] Lee KH, Gordon A, Shedden K, Kuan G, Ng S, Balmaseda A, et al. The respiratory microbiome and susceptibility to influenza virus infection. PloS ONE. 2019;14(1). 10.1371/journal.pone.0207898 PMC6326417 30625134

[186] Fouhy F, Guinane CM, Hussey S, Wall R, Ryan CA, Dempsey EM (2012). High-throughput sequencing reveals the incomplete, short-term recovery of infant gut microbiota following parenteral antibiotic treatment with ampicillin and gentamicin. Antimicrob Agents Chemother.

[187] Lee KH, Foxman B, Kuan G, López R, Shedden K, Ng S (2019). The respiratory microbiota: associations with influenza symptomatology and viral shedding. Ann Epidemiol.

[188] Lu H, Zhang C, Qian G, Hu X, Zhang H, Chen C (2014). An analysis of microbiota-targeted therapies in patients with avian influenza virus subtype H7N9 infection. BMC Infect Dis.

[189] Bandoro C, Runstadler J, Bandoro C, Runstadler J (2017). Bacterial lipopolysaccharide destabilizes influenza viruses. MSphere.

[190] Chen H-W, Liu P-F, Liu Y-T, Kuo S, Zhang X-Q, Schooley RT (2016). Nasal commensal *Staphylococcus epidermidis *counteracts influenza virus. Sci Rep.

[191] Steed AL, Christophi GP, Kaiko GE, Sun L, Goodwin VM, Jain U (2017). The microbial metabolite desaminotyrosine protects from influenza through type I interferon. Science.

[192] Pauli E-K, Schmolke M, Wolff T, Viemann D, Roth J, Bode JG (2008). Influenza A virus inhibits type I IFN signaling via NF-κB-dependent induction of SOCS-3 expression. PLoS Pathog..

[193] Belkacem N, Serafini N, Wheeler R, Derrien M, Boucinha L, Couesnon A (2017). *Lactobacillus paracasei *feeding improves immune control of influenza infection in mice. PloS ONE..

[194] Yasui H, Kiyoshima J, Hori T, Shida K (1999). Protection against influenza virus infection of mice fed Bifidobacterium breve YIT4064. Clin Diagn Lab Immunol.

[195] Chen C-J, Wu G-H, Kuo R-L, Shih S-R (2017). Role of the intestinal microbiota in the immunomodulation of influenza virus infection. Microbes Infect.

[196] Sareneva T, Matikainen S, Kurimoto M, Julkunen I (1998). Influenza A virus-induced IFN-α/β and IL-18 synergistically enhance IFN-γ gene expression in human T cells. J Immunol.

[197] Wang J, Li F, Sun R, Gao X, Wei H, Li L-J (2013). Bacterial colonization dampens influenza-mediated acute lung injury via induction of M2 alveolar macrophages. Nat Commun.

[198] Rosshart SP, Vassallo BG, Angeletti D, Hutchinson DS, Morgan AP, Takeda K (2017). Wild mouse gut microbiota promotes host fitness and improves disease resistance. Cell.

[199] Perrin-Cocon L, Aublin-Gex A, Sestito SE, Shirey KA, Patel MC, André P (2017). TLR4 antagonist FP7 inhibits LPS-induced cytokine production and glycolytic reprogramming in dendritic cells, and protects mice from lethal influenza infection. Sci Rep.

[200] Georgel A-F, Cayet D, Pizzorno A, Rosa-Calatrava M, Paget C, Sencio V (2019). Toll-like receptor 5 agonist flagellin reduces influenza A virus replication independently of type I interferon and interleukin 22 and improves antiviral efficacy of oseltamivir. Antiviral Res.

[201] Hajam IA, Kim J, Lee JH (2019). Intranasally administered polyethylenimine adjuvanted influenza M2 ectodomain induces partial protection against H9N2 influenza A virus infection in chickens. Vet Immunol Immunopathol.

[202] de Steenhuijsen Piters WA, Heinonen S, Hasrat R, Bunsow E, Smith B, Suarez-Arrabal M-C (2016). Nasopharyngeal microbiota, host transcriptome, and disease severity in children with respiratory syncytial virus infection. Am J Respir Crit Care Med.

[203] Persia S, Frassanito A, Nenna R, Petrarca L, Di Mattia G, Merola A, et al. Nasal microbiota in RSV microbiota. Eur Respiratory Soc; 2019.

[204] Teo SM, Tang HH, Mok D, Judd LM, Watts SC, Pham K, et al. Dynamics of the upper airway microbiome in the pathogenesis of asthma-associated persistent wheeze in preschool children. BioRxiv. 2017:222190.

[205] Kanmani P, Clua P, Vizoso-Pinto MG, Rodriguez C, Alvarez S, Melnikov V (2017). Respiratory commensal bacteria *Corynebacterium pseudodiphtheriticum *improves resistance of infant mice to respiratory syncytial virus and *Streptococcus pneumoniae *superinfection. Front Microbiol.

[206] Munoz FM, Englund JA (2020). Respiratory syncytial virus.&nbsp;Maternal immunization.

[207] Heldin P, Lin C-Y, Kolliopoulos C, Chen Y-H, Skandalis SS (2019). Regulation of hyaluronan biosynthesis and clinical impact of excessive hyaluronan production. Matrix Biol.

[208] Hsu P-C, Chiou B-H, Huang C-M (2018). On revealing the gene targets of Ebola virus microRNAs involved in the human skin microbiome. PeerJ.

[209] Hsu P-C, Chiou B-H, Huang C-M (2017). Bioinformatics approach reveals the gene targets of Ebola virus microRNAs involved in the human skin microbiome. PeerJ Preprints.

[210] Lozupone CA, Li M, Campbell TB, Flores SC, Linderman D, Gebert MJ (2013). Alterations in the gut microbiota associated with HIV-1 infection. Cell Host Microbe.

[211] Dang AT, Cotton S, Sankaran-Walters S, Li C-S, Lee C-YM, Dandekar S (2012). Evidence of an increased pathogenic footprint in the lingual microbiome of untreated HIV infected patients. BMC Microbiol.

[212] Mutlu EA, Keshavarzian A, Losurdo J, Swanson G, Siewe B, Forsyth C (2014). A compositional look at the human gastrointestinal microbiome and immune activation parameters in HIV infected subjects. PLoS Pathog..

[213] Mukherjee PK, Chandra J, Retuerto M, Sikaroodi M, Brown RE, Jurevic R (2014). Oral mycobiome analysis of HIV-infected patients: identification of Pichia as an antagonist of opportunistic fungi. PLoS Pathog..

[214] Ahmed N, Daniel B, Varghese J, Evangeline R, Jose T (2020). Oropharyngeal microbiome of an HIV-positive patient. Microb Pathog.

[215] Jasinska AJ, Dong TS, Lagishetty V, Katzka W, Jacobs JP. Shifts in microbial diversity, composition and functionality in the gut and genital microbiome during a natural SIV infection in vervet monkeys. 10.1186/s40168-020-00928-4 PMC7648414 33158452

[216] Li H, Liu X, Chen F, Zuo K, Wu C, Yan Y (2018). Avian influenza virus subtype H9N2 affects intestinal microbiota, barrier structure injury, and inflammatory intestinal disease in the chicken ileum. Viruses.

[217] Wen Z, Xie G, Zhou Q, Qiu C, Li J, Hu Q (2018). Distinct nasopharyngeal and oropharyngeal microbiota of children with influenza A virus compared with healthy children. BioMed Res Int..

[218] Chaban B, Albert A, Links MG, Gardy J, Tang P, Hill JE (2013). Characterization of the upper respiratory tract microbiomes of patients with pandemic H1N1 influenza. PloS ONE..

[219] Charoenkul K, Nasamran C, Janetanakit T, Tangwangvivat R, Bunpapong N, Boonyapisitsopa S (2020). Human Norovirus Infection in Dogs, Thailand. Emerg Infect Dis.

[220] Sullender ME, Baldridge MT (2018). Norovirus interactions with the commensal microbiota. PLoS Pathog..

[221] Nelson AM, Elftman MD, Pinto AK, Baldridge M, Hooper P, Kuczynski J (2013). Murine norovirus infection does not cause major disruptions in the murine intestinal microbiota. Microbiome.

[222] Nelson AM, Walk ST, Taube S, Taniuchi M, Houpt ER, Wobus CE (2012). Disruption of the human gut microbiota following Norovirus infection. PloS ONE.

[223] Parker EP, Praharaj I, Zekavati A, Lazarus RP, Giri S, Operario DJ (2018). Influence of the intestinal microbiota on the immunogenicity of oral rotavirus vaccine given to infants in south India. Vaccine.

[224] Li M, Monaco MH, Wang M, Comstock SS, Kuhlenschmidt TB, Fahey GC (2014). Human milk oligosaccharides shorten rotavirus-induced diarrhea and modulate piglet mucosal immunity and colonic microbiota. ISME J.

[225] Fix J, Chandrashekhar K, Perez J, Bucardo F, Hudgens MG, Yuan L (2020). Association between gut microbiome composition and rotavirus vaccine response among Nicaraguan infants. Am J Trop Med Hyg.

[226] Seeger C, Mason WS (2000). Hepatitis B virus biology. Microbiol Mol Biol Rev.

[227] Thio CL, Hawkins C, Hepatitis B, Virus (2020). Mandell, Douglas, and Bennett’s Principles and Practice of Infectious Diseases.

[228] Kang Y, Cai Y (2017). Gut microbiota and hepatitis-B-virus-induced chronic liver disease: implications for faecal microbiota transplantation therapy. J Hosp Infect.

[229] Ling Z, Liu X, Cheng Y, Jiang X, Jiang H, Wang Y (2015). Decreased diversity of the oral microbiota of patients with hepatitis B virus-induced chronic liver disease: a pilot project. Sci Rep.

[230] Acharya C, Sahingur SE, Bajaj JS (2017). Microbiota, cirrhosis, and the emerging oral-gut-liver axis. JCI Insight..

[231] Frumento D (2018). Microbiota and HCV infection interplay. CTBE B.

[232] Harris VC (2018). The significance of the intestinal microbiome for vaccinology: from correlations to therapeutic applications. Drugs.

[233] Velavan TP, Meyer CG (2020). The COVID-19 epidemic. Trop Med Int Health.

[234] Lai C-C, Shih T-P, Ko W-C, Tang H-J, Hsueh P-R. Severe acute respiratory syndrome coronavirus 2 (SARS-CoV-2) and corona virus disease-2019 (COVID-19): the epidemic and the challenges. Int J Antimicrob Agents. 2020:105924. 10.1016/j.ijantimicag.2020.105924 PMC7127800 32081636

[235] Song Y, Liu P, Shi X, Chu Y, Zhang J, Xia J, et al. SARS-CoV-2 induced diarrhoea as onset symptom in patient with COVID-19. Gut. 2020. 10.1136/gutjnl-2020-320891 32139552

[236] Shen Z, Xiao Y, Kang L, Ma W, Shi L, Zhang L, et al. Genomic diversity of SARS-CoV-2 in Coronavirus Disease 2019 patients. Clin Infecti Dis. 2020. 10.1093/cid/ciaa203 PMC7108196 32129843

[237] Lippi G, Henry BM. Chronic obstructive pulmonary disease is associated with severe coronavirus disease 2019 (COVID-19). Respir Med. 2020. 10.1016/j.rmed.2020.105941 PMC7154502 32421537

[238] Perlot T, Penninger JM (2013). ACE2–From the renin–angiotensin system to gut microbiota and malnutrition. Microbes Infect.

[239] Feng Z, Wang Y, Qi W. The Small Intestine, an Underestimated Site of SARS-CoV-2 Infection: From Red Queen Effect to Probiotics. 2020.

[240] Fanos V, Pintus MC, Pintus R, Marcialis MA (2020). Lung microbiota in the acute respiratory disease: from coronavirus to metabolomics. J Pediatric Neonatal Individ Med.

[241] Gheblawi M, Wang K, Viveiros A, Nguyen Q, Zhong J-C, Turner AJ (2020). Angiotensin converting enzyme 2: SARS-CoV-2 receptor and regulator of the renin-angiotensin system. Circ Res..

[242] Gao QY, Chen YX, Fang JY (2020). 2019 Novel coronavirus infection and gastrointestinal tract. J Dig Dis..

[243] Liu Y, Chan W, Wang Z, Hur J, Xie J, Yu H, et al. Ontological and bioinformatic analysis of anti-coronavirus drugs and their implication for drug repurposing against COVID-19. 2020.

[244] Anderson G. Psychological Stress and Covid-19: Interactions with gut microbiome and circadian rhythm in driving symptom severity. 2020.

[245] Gao J, Xu K, Liu H, Liu G, Bai M, Peng C (2018). Impact of the gut microbiota on intestinal immunity mediated by tryptophan metabolism. Front Cell Infect Microbiol.

[246] Markus RP, Fernandes PA, Kinker GS, da Silveira Cruz-Machado S, Marçola M (2018). Immune‐pineal axis–acute inflammatory responses coordinate melatonin synthesis by pinealocytes and phagocytes. Br J Pharmacol.

[247] Meazzi S, Lauzi S, Stranieri A, Paltrinieri S, Giordano A. The gut microbiome and mucosal defenses in cats with coronaviruses: a pilot study. Int J Health Anim Sci Food Saf. 2017;4(1 s).

[248] Sheng W-H, Chiang B-L, Chang S-C, Ho H-N, Wang J-T, Chen Y-C (2005). Clinical manifestations and inflammatory cytokine responses in patients with severe acute respiratory syndrome. J Formosan Med Assoc.

[249] Ramanathan K, Antognini D, Combes A, Paden M, Zakhary B, Ogino M (2020). Planning and provision of ECMO services for severe ARDS during the COVID-19 pandemic and other outbreaks of emerging infectious diseases. Lancet Respir Med..

[250] Mukherjee S, Hanidziar D, Focus (2018). Nutrition and food science: more of the gut in the lung: How two microbiomes meet in ARDS. Yale J Biol Med.

[251] Zhang B, Chassaing B, Shi Z, Uchiyama R, Zhang Z, Denning TL (2014). Prevention and cure of rotavirus infection via TLR5/NLRC4–mediated production of IL-22 and IL-18. Science.

[252] Gewirtz AT, Zhang B. TLR5 ligands, therapeutic methods, and compositions related thereto. Google Patents; 2018.

[253] Golonka RM, Saha P, Yeoh BS, Chattopadhay S, Gewirtz AT, Joe B (2020). Harnessing innate immunity to eliminate SARS-CoV-2 and ameliorate COVID-19 disease.

[254] Oh JZ, Ravindran R, Chassaing B, Carvalho FA, Maddur MS, Bower M (2014). TLR5-mediated sensing of gut microbiota is necessary for antibody responses to seasonal influenza vaccination. Immunity.

[255] Yu F-s, Cornicelli MD, Kovach MA, Newstead MW, Zeng X, Kumar A (2010). Flagellin stimulates protective lung mucosal immunity: role of cathelicidin-related antimicrobial peptide. J Immunol.

[256] Benedikz EK, Bailey D, Cook CN, Gonçalves-Carneiro D, Buckner MM, Blair JM (2019). Bacterial flagellin promotes viral entry via an NF-kB and Toll Like Receptor 5 dependent pathway. Sci Rep.

[257] Müller H, Dagher G, Loibner M, Stumptner C, Kungl P, Zatloukal K (2020). Biobanks for life sciences and personalized medicine: importance of standardization, biosafety, biosecurity, and data management. Curr Opin Biotechnol.

